# Standardization of esophageal adenocarcinoma in vitro model and its applicability for model drug testing

**DOI:** 10.1038/s41598-021-85530-w

**Published:** 2021-03-23

**Authors:** Larisa Tratnjek, Nadica Sibinovska, Slavko Kralj, Darko Makovec, Katja Kristan, Mateja Erdani Kreft

**Affiliations:** 1 Faculty of Medicine, Institute of Cell Biology, University of Ljubljana, Vrazov trg 2, 1000 Ljubljana, Slovenia; 2 Faculty of Pharmacy, Chair of Biopharmaceutics and Pharmacokinetics, University of Ljubljana, Aškerčeva c. 7, 1000 Ljubljana, Slovenia; 3 Department for Materials Synthesis, Jožef Stefan Institute, Jamova 39, 1000 Ljubljana, Slovenia; 4 Lek Pharmaceuticals, d.d., Sandoz Development Center Slovenia, Verovškova 57, 1526 Ljubljana, Slovenia

**Keywords:** Biological techniques, Cancer, Cell biology, Drug discovery, Diseases, Oncology, Engineering

## Abstract

FLO-1 cell line represents an important tool in esophageal adenocarcinoma (EAC) research as a verified and authentic cell line to study the disease pathophysiology and antitumor drug screenings. Since in vitro characteristics of cells depend on the microenvironment and culturing conditions, we performed a thorough characterization of the FLO-1 cell line under different culturing conditions with the aim of (1) examining the effect of serum-free growth medium and air–liquid interface (A–L) culturing, which better reflect physiological conditions in vivo and (2) investigating the differentiation potential of FLO-1 cells to mimic the properties of the in vivo esophageal epithelium. Our study shows that the composition of the media influenced the morphological, ultrastructural and molecular characteristics of FLO-1 cells, such as the expression of junctional proteins. Importantly, FLO-1 cells formed spheres at the A–L interface, recapitulating key elements of tumors in the esophageal tube, i.e., direct contact with the gas phase and three-dimensional architecture. On the other hand, FLO-1 models exhibited high permeability to model drugs and zero permeability markers, and low transepithelial resistance, and therefore poorly mimicked normal esophageal epithelium. In conclusion, the identified effect of culture conditions on the characteristics of FLO-1 cells should be considered for standardization, data reproducibility and validity of the in vitro EAC model. Moreover, the sphere-forming ability of FLO-1 cells at the A–L interface should be considered in EAC tumor biology and anticancer drug studies as a reliable and straightforward model with the potential to increase the predictive efficiency of the current in vitro approaches.

## Introduction

Esophageal Adenocarcinoma (EAC) shows the steepest rise in incidence in Western countries over recent years than any other cancer, due to increased risk factors such as gastroesophageal reflux, smoking, and obesity^1–6^. The clinical outcome, however, remains poor. EAC is a highly aggressive and highly mutated cancer with a high degree of heterogeneity^7–9^. Main problems for the treatment of EAC presents late detection (the majority of patients presents with EAC at a late stage with advanced or metastatic disease)^1,5,10^, and resistance to conventional chemotherapy ^5^. Consequently, there is a need to identify highly efficient novel molecular targeted treatments.

EAC remains one of the least studied malignancies with the lack of model systems to study pathogenesis and drug testing. Cell lines are an important tool in EAC research as they enable investigation of molecular pathways involved in EAC tumorigenesis and metastasis, and development and testing of anti-cancer therapies. They are easy to grow and manipulate in vitro and in animal xenograft models^11,12^. An important advantage of cell lines in cancer research is controllable experimental variables and quantitative analysis, which are difficult to extract in living system^12^. Furthermore, in vitro methods are an important alternative for animal experiments^13^. Currently, there are no widely accepted animal models of EAC. FLO-1 cell line is an authentic, verified, non-contaminated cell line derived from human EAC and as such allows for the replacement, reduction and refinement (3Rs) in laboratory animal use. Its identity has been verified by short tandem repeat analysis, p53 mutation and xenograft histology against the original tumors^11^. The whole-genome sequencing of FLO-1 cell line confirmed the presence of many of the known mutations that drive esophageal cancer^7^. FLO-1 develops spontaneous metastases after subcutaneous inoculation in mice, while their location mirrored those seen in EAC patients (tumors predominately present in the lung, liver, peritoneum and mediastinal lymph nodes)^14^, validating the utility of FLO-1 cell as a preclinical model of metastasis in EAC. Moreover, as one of the EAC cell lines, FLO-1 cells are used as in vitro model for disease pathophysiology, high throughput analysis and selection of potential drugs for treatment, cytotoxic and synergistic studies, and to examine the antitumor effect of drugs^15–19^.

Despite the widespread use of FLO-1 cell line as an in vitro model of EAC, a comprehensive morphological and ultrastructural characterization with respect to culture conditions has not yet been performed. Since culture conditions significantly influence the characteristics of the cultivated cells, the optimization of experimental conditions is crucial to ensure robust experimental consistency and reproducibility. Furthermore, the culture conditions must reflect the physiological conditions in order to increase the validity of in vitro model. Therefore, in this study, we have maintained FLO-1 cells under culture conditions which better reflect in vivo conditions and compared them to FLO-1 in vitro models maintained under standard conditions. We have examined the effect of (1) low serum and serum-free growth medium, (2) air–liquid (A–L) culturing interface, and (3) post-confluent long-term culturing.

Namely, the Guidelines on good cell culture practice and the ECVAM Scientific Advisory Committee (ESAC, 2008) recommend the use of serum-free media^20–22^, as serum carries a potential risk of contamination and batch-to-batch variability. Furthermore, serum supplementation does not represent physiological conditions and may alter the experimental output or assay^23^. FLO-1 cells are routinely cultured in medium containing 10% serum^14–16,18,19^; therefore we have tested their growth in low-serum and serum-free media.

Next, we have investigated the effect of A–L interface culturing, as it was shown that the A–L approach supports the maintenance of in vivo-like functionality of many epithelial cells, including the esophagus-derived cells^24,25^ and the fact that air is present in the esophageal tube^26,27^. In the A–L culturing system, the cells grow on the porous membrane with the apical cell surface exposed to air and the basal surface is in contact with the growth medium, unlike the liquid–liquid (L–L) interface culturing system where both surfaces are in contact with the growth medium. Next, while most studies keep FLO-1 cells in vitro for short periods of time^18,19,28^, it has been shown that post-confluent culturing of cancer cells results in cultures that exhibit many of the properties of solid tumors^29–32^. We kept FLO-1 cells in vitro for different periods of time: 1, 2 and 4 weeks (wks) and compared the results.

In addition, we investigated the differentiation potential of FLO-1 cells in vitro, as culture models of several cancer cell lines were developed that form a tight barrier, and/or partially mimic the properties of certain epithelia in vivo^33–38^.

The effect of different culturing conditions was evaluated by ultrastructural analysis of the cells with scanning electron microscopy (SEM) and transmission electron microscopy (TEM), immunolabeling of cell junction proteins, transepithelial resistance (TER) measurements, and model drugs and zero permeability markers permeability studies.

## Materials and methods

### Cell culture

The FLO-1 cells were obtained from the Public Health England, European Collection of Authenticated Cell Cultures (ECACC General Cell Collection; Cat. No. 11012001, lot No. 15B014, passage number 7, STR verification: 24/03/2015, PCR-based mycoplasma detection: 13/03/2015) and used from passage 10 to 17. The cells were maintained in 3 different culture media; (1) DMEM (Dulbecco's Modified Eagle Medium with GlutaMAX (4 mM), 4.5 g/L glucose and 25 mM HEPES, Gibco, ThermoFisher, Waltham, MA, USA, Cat. No 32430-027) supplemented with 10% Fetal Bovine Serum (FBS, Gibco, Cat. No. 10108-165) and 1 mM sodium pyruvate (Gibco, Cat. No. 11360), (2) A-DMEM (Advanced Dulbecco’s Minimum Essential Medium, Gibco, Cat. No. 12491-015) supplemented with 4 mM GlutaMAX (ThermoFisher, Cat. No. 35050–038) and 2.5% FBS, (3) UroM (previously used also for other epithelial cell types^39–43^), consisting of equal parts of MCDB153 medium (Sigma-Aldrich, Taufkirchen, Germany, Cat. No. M 7403) and Advanced-Dulbecco’s modified essential medium (Gibco, Cat. No. 12491–015) supplemented with 0.1 mM phosphoethanolamine (Sigma-Aldrich, Cat. No. P 0503), 15 µg/ml adenine (Sigma-Aldrich, Cat. No. A 2786), 0.5 µg/ml hydrocortisone (Sigma-Aldrich, Cat. No. H 0888), 5 µg/ml insulin (Sigma-Aldrich, Cat. No. I 1882), 4 mM GlutaMAX and with either 2.5% FBS (0.9 mM extracellular calcium concentration) for the first week of culturing or with 2.5 mM calcium (CaCl_2_, UKC Ljubljana, Slovenia) for additional 1–3 wks (serum-free UroM).

The cell cultures were maintained at 37 °C in a > 95% humidified atmosphere of 5% CO_2_ in the air with media changes on alternate days. Cultured cells were observed daily under a phase-contrast microscope (DM-IL Leica (Leica Microsystems GmbH, Wetzlar, Germany), objective L40 × /0.50 Ph2 and Nikon Eclipse TE300 (Melville, NY, USA), objective DIC Plan Fluoro L10 × /0.30 Ph1).

Once 70–80% confluent, the cells were harvested. The cells were detached with TrypLE Select (Gibco), collected and centrifuged at 200 g for 5 min at room temperature. The cell pellet was then resuspended in culture medium, cells were counted with a haemocytometer and their viability was assessed with the Trypan blue dye (Gibco) exclusion method.

For the evaluation of FLO-1 cell models on different substrates and interfaces while maintained in three different media types, the cells were seeded at a density of 3 × 10^4^ cells/cm^2^ (according to the ECACC cell collection, recommended seeding density is 1–3 × 10^4^ cells/cm^2^) onto polystyrene Tissue Culture Flasks, Cell culture 9.2 cm^2^ dishes (TPP, Trasadingen, Switzerland), 96-microplates (Microplate 96-W, PS, TC, Black/clear B, BD Falcon, Bedford, MA, USA, Cat. No. 34-115-5002-000) and glass coverslips. For successful culturing of FLO-1 cells on Polyethylene Terephthalate (PET) porous membranes (BD Falcon, Cat. No. 353180), the seeding density should be higher (6 × 10^4^ cells/cm^2^) (Supplemental Fig. S1). To establish the liquid–liquid (L–L) growth interface, an appropriate volume of culture medium was added to both the apical and basal compartments of porous membrane inserts, whereas for the air–liquid (A–L) interface, the culture medium was removed from the apical compartment 24 h after the seeding. The cells were maintained in culture for 1, 2, or 4 wks.

### Cell viability

FLO-1 cells were seeded onto polystyrene Tissue Culture Flasks and maintained in DMEM, A-DMEM and UroM media. Subculturing was performed every 3–5 days and the viability of FLO-1 models was assessed with a haemocytometer and Trypan blue dye exclusion staining. Viability of FLO-1 models was also assessed with ATP measurements (CellTiter-Glo, Luminiscent Cell Viability Assay, Promega, Madison, WI, USA). The cells were seeded onto 96-microplates (Microplate 96-W, PS, TC, Black/clear B, BD Falcon, Cat. No. 34-115-5002-000) and maintained in D-MEM, A-DMEM or UroM for 1 and 2 wks. The experiment was performed for luminometric measurement of cell growth (viability) according to the standard protocol of the manufacturer. Briefly, 100 μL of the reagent was added to the 100 μL of medium-containing wells of 96-well plate. The reaction mixture was incubated at room temperature in the dark for 30 min to allow for whole microtissue lysis. The samples were then analyzed using a microplate reader Tecan Safire II (Tecan Safire2, Tecan Group Ltd, Männerdorf, Switzerland), which measured the amount of light generated by chemical reaction (luminescence), and produced a result expressed in arbitrary relative light units (RLUs). The intensity of the light is proportional to the amount of ATP in the sample.

### Transepithelial electrical resistance measurements

When the FLO-1 cells, maintained in DMEM and A-DMEM at the L–L and A–L interfaces, reached confluence on the porous membranes, TER was measured using an epithelial voltohmmeter (EVOM & EVOMX, Sarasota, Center Boulevard, FL, USA) with EndOhm-12 chamber. To conduct the measurements, 0.4 mL of fresh culture medium was added to the apical compartment of both, the A–L and L–L interface cultures for 3 min. Then the cell culture inserts were transferred into the EndOhm-12 chamber. TER was measured 5 days per week for 1 month. The measured TER values were corrected by subtracting the mean resistance of blank porous membranes [150 Ωcm^2^]. Results are expressed as the resistance of a unit area Ωcm^2^ (resistance (Ω) x effective membrane area (cm^2^)).

### Immunocytochemistry

Indirect immunofluorescence was performed as previously described^44^. Cells grown on glass coverslips for 1 and 2 wks and porous membranes at the A–L and L–L interface for 1, 2 and 4 wks in DMEM, A-DMEM and UroM media were fixed at room temperature with cold (− 20 °C) 100% ethanol for 25 min. After fixation, cells were washed with PBS and incubated in blocking solution containing 1% Bovine Serum Albumin (BSA, Sigma) in PBS for 45 min at room temperature. Primary and secondary antibodies were diluted in 1% BSA in PBS and incubated overnight at 4 °C and 1.5 h at room temperature, respectively. Extensive washing with PBS was performed between each step. Finally, cells were mounted in DAPI-Vectashield medium (Vector Laboratories, Burlingame, CA, USA). For negative controls, incubation with primary antibodies was omitted.

### Antibodies

Primary and secondary antibodies were used at the following dilutions: Mouse monoclonal anti- cytokeratin 7 (Dako, Agilent technologies, CA, USA, Cat. No. M7018), 1:20; Mouse monoclonal anti-E-cadherin (BD Transduction Laboratories, San Jose, CA, USA; Cat. No. 610182), 1:30; Rabbit polyclonal anti-N-cadherin (Abcam, Cambridge, MA, USA; Cat. No. ab18203), 1:100; Rabbit polyclonal anti-occludin (Invitrogen, Thermo Fisher Scientific, San Francisco, CA, USA, Cat. No. 71-1500), 1:30; Mouse monoclonal anti-claudin-2 (Zymed Laboratories, Thermo Fisher Scientific, Cat. No. 32-5600), 1:100; Rabbit polyclonal anti-claudin-4 (Zymed Laboratories, Thermo Fisher, Cat. No. 32-9400), 1:100; Alexa-Fluorophore-conjugated anti-mouse and anti-rabbit secondary antibodies (Invitrogen, Molecular Probes, Leiden, The Netherlands), 1:400.

### Fluorescence microscopy

3D images were obtained using the fluorescence microscope AxioImager.Z1 with an Apotome attachment for optical sectioning (Carl Zeiss MicroImagingGmbh, Heidelberg, Germany; 63X oil immersion objective, numerical aperture 1.4, image size 1388 × 1040 pixels, 14.435 μm^2^, pixel size 0.1 μm). Z axis step was set to 0.280 µm.

### Transmission electron microscopy

Transmission electron microscopy was carried out as previously described^40,45^. FLO-1 cells cultured at the A–L and L–L interfaces on porous membranes (BD Falcon) for 1, 2 or 4 wks and cell culture dishes for 1 or 2 wks in DMEM, A-DMEM and UroM were fixed with 4% (w/v) formaldehyde (Sigma) and 2.5% (v/v) glutaraldehyde (Serva, Heidelberg, Germany) in 0.1 M cacodylate buffer, pH 7.4 for 2 h 45 min. The fixation was followed by overnight rinsing in 0.1 M cacodylate buffer and post-fixation in 2% (w/v) osmium tetroxide for 1 h at room temperature. Afterwards, the samples were incubated in 2% uranyl acetate (Merck, Germany) for 1 h at room temperature. The samples were then dehydrated in a graded series of ethanol and embedded in Epon (Serva). Epon semi-thin sections were stained with 1% toluidine blue and 2% borate in distilled water and observed with a Nicon Eclipse TE microscope. Ultrathin sections were contrasted with uranyl acetate and lead citrate and observed with a transmission electron microscope (Philips CM100, (Philips, Eindhoven, The Netherlands), operation voltage 80 kV, equipped with CCD camera (AMT, Danvers, MA, USA)).

### Scanning electron microscopy

Scanning electron microscopy was carried out as previously described^35,41,45,46^. FLO-1 cells cultured at the A–L and L–L interfaces on porous membranes (BD Falcon) in DMEM, A-DMEM and UroM for 1, 2 or 4 wks were fixed in 2% formaldehyde (w/v) and 2% glutaraldehyde (v/v) in 0.1 M cacodylate buffer, pH 7.4 for 2 h 45 min at 4 °C. The fixation was followed by rinsing in 0.2 M cacodylate buffer. The samples were then post-fixed in 1% (w/v) osmium tetroxide for 2 h at room temperature, dehydrated in a graded series of ethanol, dried at a critical point, spattered with gold and examined at 30 kV with a Tescan Vega3 scanning electron microscope (Brno, Czech Republic).

### Permeability of fluorescent iNANO-RITC nanoparticles

FLO-1 cells were seeded onto porous membranes (Merck Millipore, Tullagreen, Ireland, Cat No. PSMW010R5 and PSRP010R5 (pore size 1 μm), and BD Falcon, Cat. No. 353180 (pore size 0.4 μm)) and cultured at the A–L and L–L interfaces in A-DMEM for 1 wk. The permeability through all cell layers of the A–L and L–L FLO-1 models was assessed using the fluorescent core–shell iNANO-RITC nanoparticles (“iNANOvative|BIO-RITC silica” commercially available by Nanos Scientificae d.o.o, Ljubljana, Slovenia). The mean size of iNANO-RITC nanoparticles is 100 nm containing 70 nm-sized iron oxide core and a 15 nm thick silica shell ^47,48^. Rhodamine B fluorescent dye was covalently bonded with alkoxy silane molecule and integrated inside primary 5-nm-thick silica layer^49,50^. Then, an additional 10-nm-thick layer of dye-free silica was deposited on the surface of fluorescent silica coating. Estimated mean weight of iNANO-RITC nanoparticles is 4.1 * 10^–15^ g^51,52^. After 1 wk in culture, 200 μL of 100 μg/mL iNANO RITC diluted in culture medium was added to the apical compartment for 3 h, whilst 800 μL of fresh culture medium was added to the basal compartment. The amount/fluorescence of iNANO-RITC in the apical culture surface and basal chamber was determined using a microtiter plate reader (Tecan Group Ltd, Männerdorf, Switzerland). Additionally, following the same protocol, permeability studies were performed on highly differentiated normal urothelial cells (NPU) grown for 3 wks in UroM (supplemented with 2.5% FBS just for the first 7 days) and compared to FLO-1 cells grown at A-DMEM medium, both maintained at the L–L interface. After the permeability was measured, NPU and FLO-1 cultures were fixed in 4% formaldehyde (w/v) for 15 min. The cells were then washed in PBS for 30 min and mounted onto slides with DAPI-Vectashield. The samples were examined with the AxioImager.ZI microscope with an Apotome attachment. The red channel values corresponded to the intensity of the fluorescence of endocytosed iNANO-RITC nanoparticles. Four random images were taken (image size 1388 × 1040 pixels, 0.32 µm/pixel, 147.8 µm^2^) in all samples and the average intensity of red fluorescence was measured using AxioVision 4.8 software. The results are given in arbitrary units (a. u.).

### Permeability of model drugs and zero permeability markers

For permeability assays with model drugs and zero permeability markers, the FLO-1 cells were seeded at a density of 3 × 10^4^ cells/cm^2^ on polyethylene terephthalate (PET) membranes (1 μm pores, 0.7 cm^2^) in Millicell 24-well cell culture plates (Merck Millipore, Tullagreen, Ireland). The cells were cultured in two media: DMEM and A-DMEM at the L–L interface. In order to establish L–L interface, adequate volumes of cell culture medium were added to the apical (400 μL medium/insert) and basolateral compartments (22–24 mL medium/feeder tray). FLO-1 cells were cultured for 1 wk. Prior to conducting the permeability assay, the FLO-1 cells were rinsed twice with assay buffer consisting of Hank’s balanced salt solution (HBSS) (Thermo Fisher Scientific, Waltham, MA, USA) and 0.01 M HEPES (Thermo Fisher Scientific, Waltham, MA, USA), pre-warmed at 37 °C.

Permeability assays with model drugs and zero permeability markers were carried out as previously described^53^. Model drugs, zero permeability markers and chemicals were obtained from Sigma-Aldrich (Munich, Germany). All solutions of the model drugs and zero permeability markers were prepared in assay buffer (HBSS with 0.01 M HEPES). When the desired concentrations of the model drugs could not be achieved due to low solubility of the compounds, assay buffer with 1% DMSO (Sigma-Aldrich (Munich, Germany)) was used. The investigated model drugs and zero permeability markers and the tested concentrations are shown in Table 1. For the transport studies in apical to basolateral (A-B) direction, 400 μL of pre-warmed test solutions and 800 μL of pre-warmed assay buffer were added to the apical and basolateral compartments, respectively. When assessing permeability in basolateral to apical (B-A) direction, 400 μL of pre-warmed assay buffer was added to the apical and 800 μL of pre-warmed test solutions were added to the basolateral compartment. The 24-well cell culture plates with FLO-1 cells were incubated at 37 °C in a humidified 5%CO_2_/95% air atmosphere throughout the bidirectional permeability assays. The 100 μL samples (except for azilsartan—150 μL) were withdrawn from the acceptor wells at predetermined time points (30, 60, 90, 120, and 180 min), while 20 or 10 μL samples were withdrawn from the donor wells for the model drugs or zero permeability markers, respectively, at 0 and 180 min. The withdrawn volume was replenished with a fresh assay buffer or donor solution.

**Table 1:** Summary of doses used for testing permeability of model drugs and zero permeability markers (ZPM), dissolved in 250 mL assay buffer.

Tested substance	Tested concentration (mg/250 mL)	Permeability category based on oral absorbed fraction (f_a_)	Oral absorbed fraction f_a_ (%)^f^
Propranolol^b^	80	High	100
**Antipyrine**	50	High	99
Metoprolol^c^	100	High	95
**Losartan**^d^	50	Moderate	65
Furosemide^a^	80	Moderate	61
**Ranitidine**^b^	300	Moderate	50
Atenolol	100	Moderate	50
Famotidine^a^	40	Low	45
**Oxacillin**^e^	500	Low	33
Nadolol	160	Low	27
**Chlorothiazide**^a^	8	Low	20
**Azilsartan**^a^	19	Low^g^	No data available
FD 3–5 kDa	50	ZPM	
FD 10 kDa	50	ZPM	
FD 20 kDa	50	ZPM	
FD 40 kDa	50	ZPM	
FD 70 kDa	50	ZPM	

The permeability of all model drugs and zero permeability markers was tested in FLO-1 cells cultured in DMEM, while several model drugs (in bold) were also tested in FLO-1 cells cultured in A-DMEM.

^a^DMSO used for preparing solutions of model drugs (maximum 1% DMSO in the final solution).

^b^Tested substances used in their hydrochloride salt form.

^c^Substance tested as metoprolol tartrate.

^d^Substance tested as losartan potassium.

^e^Substance tested as oxacillin sodium monohydrate.

^f^Reference^33^.

^g^Reference^54^.

### Analytical methods

The amount of model drugs which permeated through the cell layers was quantified by ultra-high pressure liquid chromatography (UHPLC Acquity separations module equipped with photodiode array detector, Empower software, Waters, Milford, MA, USA), except for azilsartan, which was quantified by using high-pressure liquid chromatography (Waters 2695 HPLC separations module equipped with photodiode array detector, Empower software, Waters, Milford, MA, USA).

UHPLC and HPLC parameters for all tested model drugs were described previously ^53^. Quantification of the amount of the zero permeability markers, i.e. FITC-dextrans (FDs) with molecular weights (MW) of 3–5 kDa, 10 kDa, 20 kDa, 40 kDa and 70 kDa, was done by measuring the fluorescence intensity at the appropriate excitation and emission wavelengths (λex/em = 495/515 nm), using microtiter plate reader (Infinite M1000, Tecan, Männedorf, Switzerland). The amount of diffused fluorescent compounds was calculated from a calibration curve.

### Permeability data analysis

The apparent permeability coefficients (P_app_) were calculated using the Eq. (1):

1 $${\text{P}}_{{{\text{app}}}} = {\text{k}}_{{\text{d}}} \times { }\frac{1}{{{\text{A}} \times {\text{C}}_{0} }}$$

where k_d_ (mol/s or mg/s) is the slope of the linear section in the amount or mass of investigated drug substance permeated to the acceptor side versus time plot, $A$ is the exposed surface area of the cell monolayer (0.7 cm^2^), $C_{0}$ (M or mg/mL) is the average initial concentration of drug substance in donor wells.

### Data analysis and statistics

In the study, 2–3 independent experiments were performed to examine the effect of different substrates, interfaces and different media types on in vitro characteristics of FLO-1 cell line. Within each independent experiment, 3–4 technical replicates were examined with different experimental methods. The images shown in figures are representative of at least 2 independent experiments. For TEM and SEM analysis, the high-quality micrographs were assessed independently by two of the authors. The authors had no prior knowledge about the cell culture condition, and how long and in which growth medium the cells were maintained. The calculated ATP test values, TER values, and permeability analysis values are expressed as means ± standard error (SE) of 3–24 replicates. Data were analyzed using the Graph Pad Prism program, version 8.1.2 (GraphPad Software, LaJolla, San Diego, CA, USA). Differences between experimental groups were tested for significance using un-paired two-tailed Student t-test or two-way ANOVA with Tukey’s multiple comparisons test. Statistical significance was accepted at *P* values: 0.05, 0.01, 0.001, and 0.0001, as indicated. All data are expressed as mean ± standard error (SE).

## Results

### Effect of cell culture medium type on cell proliferation

**Figure 1:**
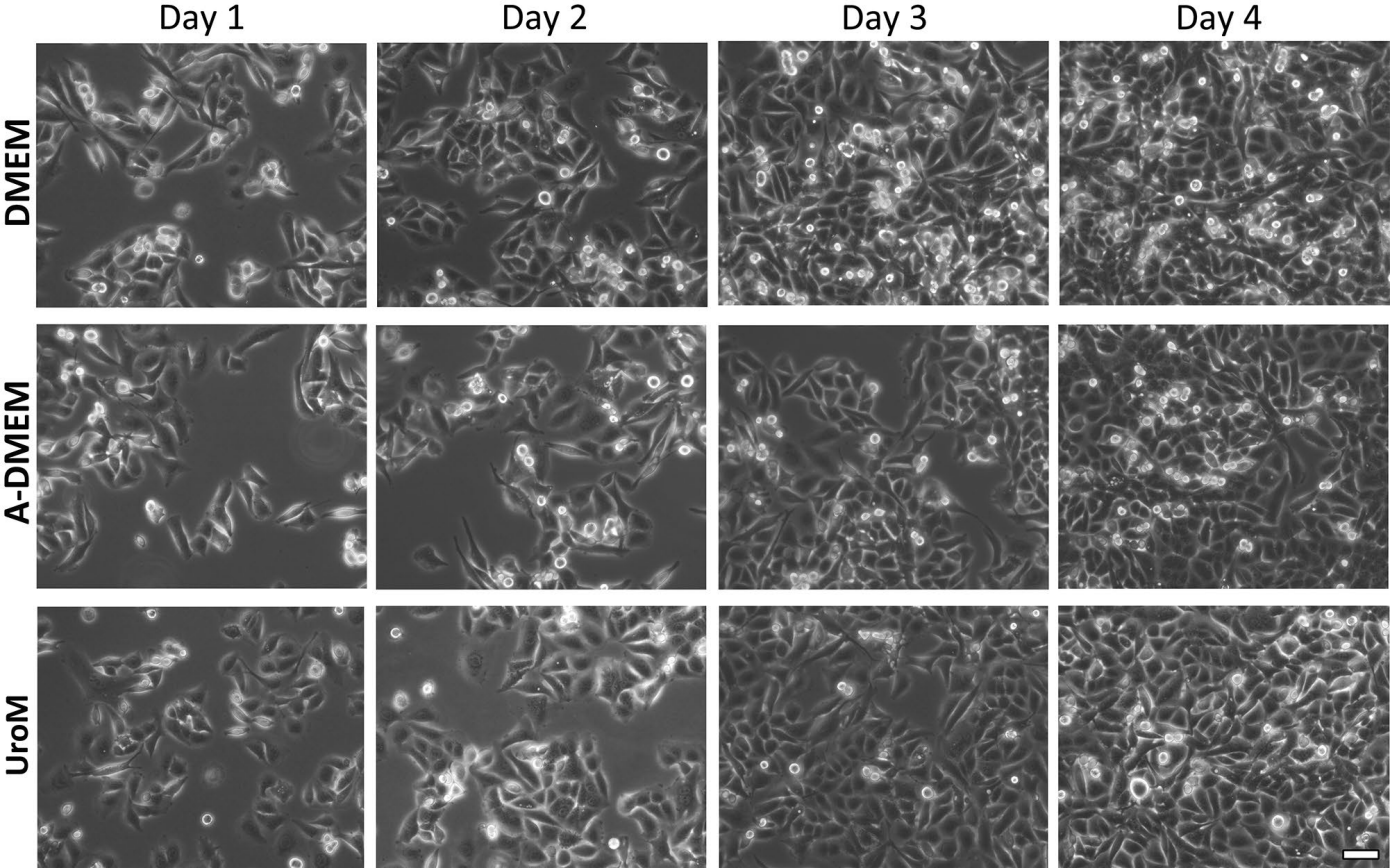
FLO-1 cell proliferation in DMEM, A-DMEM, and UroM media seeded at a density of 3 × 10^4^ cells/cm^2^ on polystyrene culture flasks on day 1–4 after the seeding, examined by live phase-contrast microscopy. Cells exhibit a polygonal shape and grow attached to a surface in discrete patches. Cells reach confluence after four days in vitro in all media types. Scale bar: 10 μm.

The esophageal adenocarcinoma cell line FLO-1 is routinely cultured in DMEM medium, supplemented with 10% FBS. In the present study, we tested the cell viability and proliferation of FLO-1 cells in two additional culture media containing a reduced FBS concentration. The A-DMEM medium was supplemented with 2.5% FBS for the entire time of culturing, while UroM medium was supplemented with 2.5% FBS only during the first 7 days. Cells were seeded onto polystyrene culture flasks at a seeding density of 3 × 10^4^ c/cm^2^. Cells exhibited polygonal shape and successfully adhered and proliferated in all culture media types. The confluence was reached after a maximum of 4 days in vitro (Fig. 1).

### Effect of culture medium type on cell viability

Cells were sub-cultured after they reached 70–80% confluence and their viability was evaluated. In all media, the determined cell viability for passaging cells was very high. The average sub-culturing viability assessed for FLO-1 cells of passages 11–17 was 97.6 ± 0.3% when maintained in DMEM medium (n = 16), 96.6 ± 0.5% in A-DMEM medium (n = 10), and 96.3 ± 1.2% in UroM medium (n = 9).

**Figure 2:**
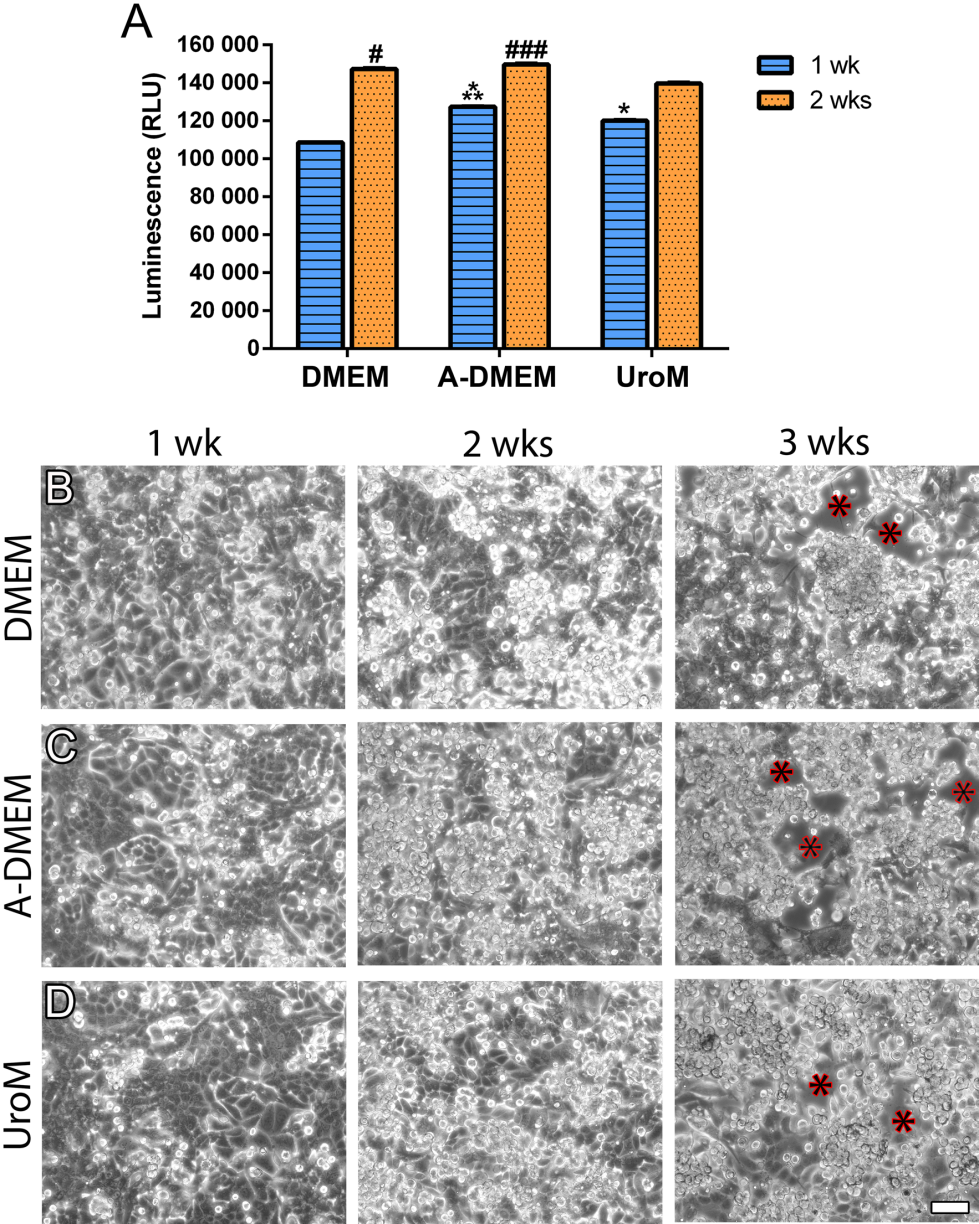
The viability of FLO-1 cells is high in all media, with FLO-1 cultures maintained in A-DMEM having the highest number of viable cells. However, with increasing culturing time, cell shedding is observed in all media types. (**A**) Cell viability was assessed by the determination of the cellular ATP level as an indirect measure of the number of viable cells. Bars represent the arbitrary relative luminescence units (RLU). Two-way ANOVA with Tukey’s multiple comparisons test was performed (n = 24 wells with cell cultures per medium type). ***After 1 wk in vitro, the number of viable cells is significantly higher in A-DMEM compared to DMEM (*p* < 0.0001) and UroM (*p* < 0.05) media. *The viability of cells maintained in UroM is significantly higher than in DMEM (*p* < 0.0001) after 1 wk. ###After 2 wks the number of viable cells is significantly higher when cells are maintained in A-DMEM compared to DMEM and UroM (*p* < 0.0001). #The viability of cells maintained in DMEM is significantly higher than in the UroM after 2 wks in culture (*p* < 0.01). (**B**) Cells detach from the growth surface (polyethylene flasks) when maintained in culture for a prolonged time (red asterisks), regardless of culture media. Scale bar, 10 µm.

The viability of FLO-1 cells maintained in vitro for 1 or 2 wks was then examined using the ATP test. After 1 wk, the cultures retained a higher number of viable cells when maintained in A-DMEM compared to DMEM and UroM media (two-way ANOVA, Tukey’s multiple comparisons test, Fig. 2A). The average number of viable FLO-1 cells was also the highest in A-DMEM medium when maintained in vitro for 2 wks and was significantly different from DMEM and UroM medium (two-way ANOVA, Tukey’s multiple comparisons test, Fig. 2A).

Daily observation of the cultures under phase-contrast microscopy revealed that FLO-1 cells detached from the growth support surface with the culture time. At 3 wks, cell shedding was observed in cultures maintained in all media types (Fig. 2B–D).

### The effect of culture medium type on the ultrastructure of the air–liquid and liquid–liquid interface FLO-1 models

Cells successfully adhered and proliferated in all types of culture media when seeded onto the porous PET membrane. FLO-1 cells were seeded onto porous membrane supports at a higher seeding density (6 × 10^4^ cells/cm^2^) as onto culture flasks since, despite higher seeding density, cells reached the confluence after 7 days (in all culture media types; Supplemental Fig. S1), whereas cells seeded onto culture flasks reached confluence after 4 days in vitro (Fig. 1). However, in cultures maintained in UroM at the A–L interface cell shedding was observed soon after reaching confluence or even before confluence was reached (Supplemental Fig. S1), whereas, in DMEM and A-DMEM medium, cell shedding started between 10 and 14 days in vitro (Supplemental Fig. S2). On the contrary, when maintained at the L–L interface in UroM medium, cell shedding was observed only in the last days of culturing (in the last, fourth week of culturing, Supplemental Fig. S2).

**Figure 3:**
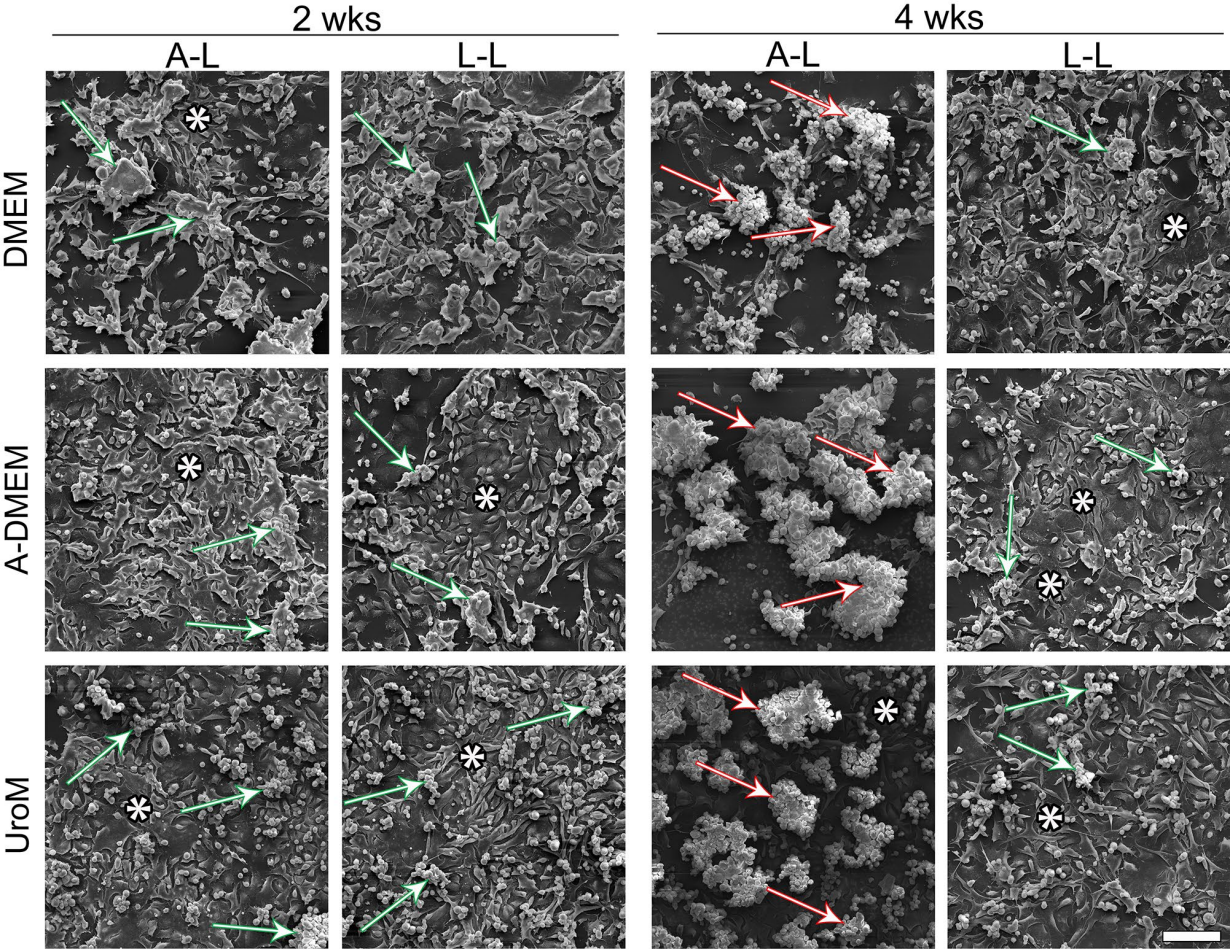
Apical surface structure of FLO-1 cells forming clusters and spheres at the A–L and L–L interfaces, respectively, surrounded by polygonal cells. FLO-1 cells were cultured on porous membranes and maintained in DMEM, A-DMEM or UroM media at the A–L and L–L interfaces for 2 or 4 wks and analyzed with low magnification SEM. Cells exhibit a polygonal morphology (asterisks) or a round morphology and form small clusters (green arrows) or large spheres (red arrows). Scale bar, 100 µm.

**Figure 4:**
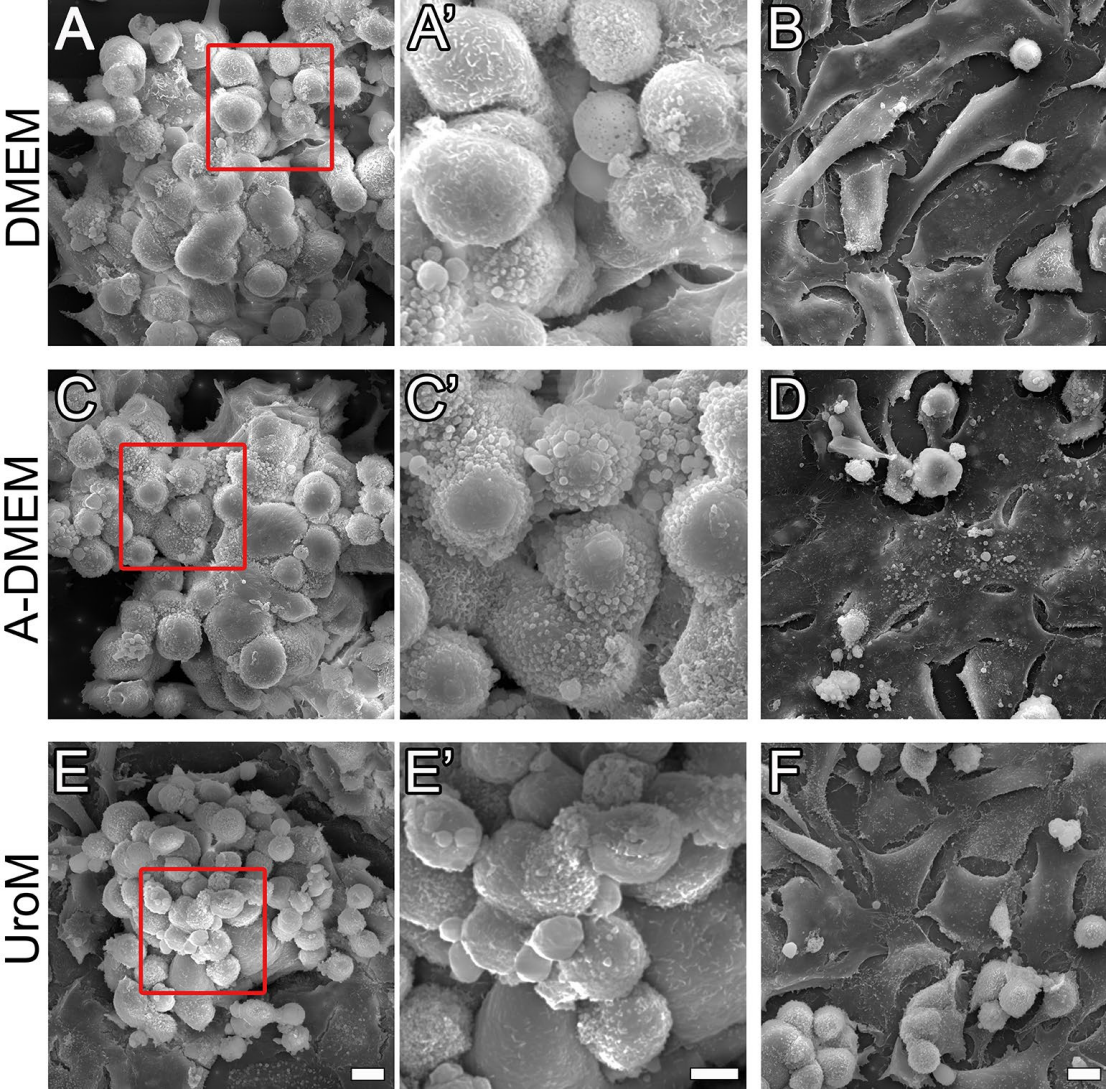
Apical surface structure of FLO-1 spheres surrounded by polygonal cells at the A–L interface. FLO-1 cells were cultured on porous membrane supports and maintained in DMEM, A-DMEM and UroM media for 4 wks and analyzed with high magnification SEM. In all media types, spheres consist of round to oval cells, which often have a rough surface (**A**, **A**’, **C**, **C**’, **E**, **E**’). Spheres are surrounded by polygonal cells, which show a smoother surface (**B**, **D**, **F**). The red boxed areas in images (**A**, **C**, and **E**) are shown in the magnified view in images (**A**’, **C**’, and **E**’), respectively. Scale bar, 10 µm (**A**,**B**,**C**,**D**,**E**,**F**), 5 µm (**A**’,**C**’,**E**’).

The cell culture architecture was interface dependent in all media types. Light microscopy and SEM showed that cells formed spheres at the A–L interface which became more numerous and larger with the culturing time (Fig. 3, Supplemental Fig. S2). After 4 wks, the diameter of the spheres reached up to 150–200 µm (Fig. 3). The spheres had a round or oval grape-like appearance, consisting of round cells with a smooth or rough surface (Fig. 4, Supplemental Fig. S3). Between the spheres, polygonal cells with tighter cell connections were present (Figs. 3, 4, Supplemental Fig. S3). Small cell clusters of round/oval cells (diameter 50 µm) were found at the L–L interface while large spheres, as observed at the A–L interface, were not formed (Fig. 3, Supplemental Fig. S3). However, similarly as spheres at the A–L interface, the cell clusters were surrounded by areas of cells with a polygonal morphology with tighter cell connections (Figs. 3, 4, Supplemental Fig. S3). Again, extensive cell shedding was observed under SEM in the cultures maintained in vitro for 4 wks in all media types and especially at the A–L interface.

**Figure 5:**
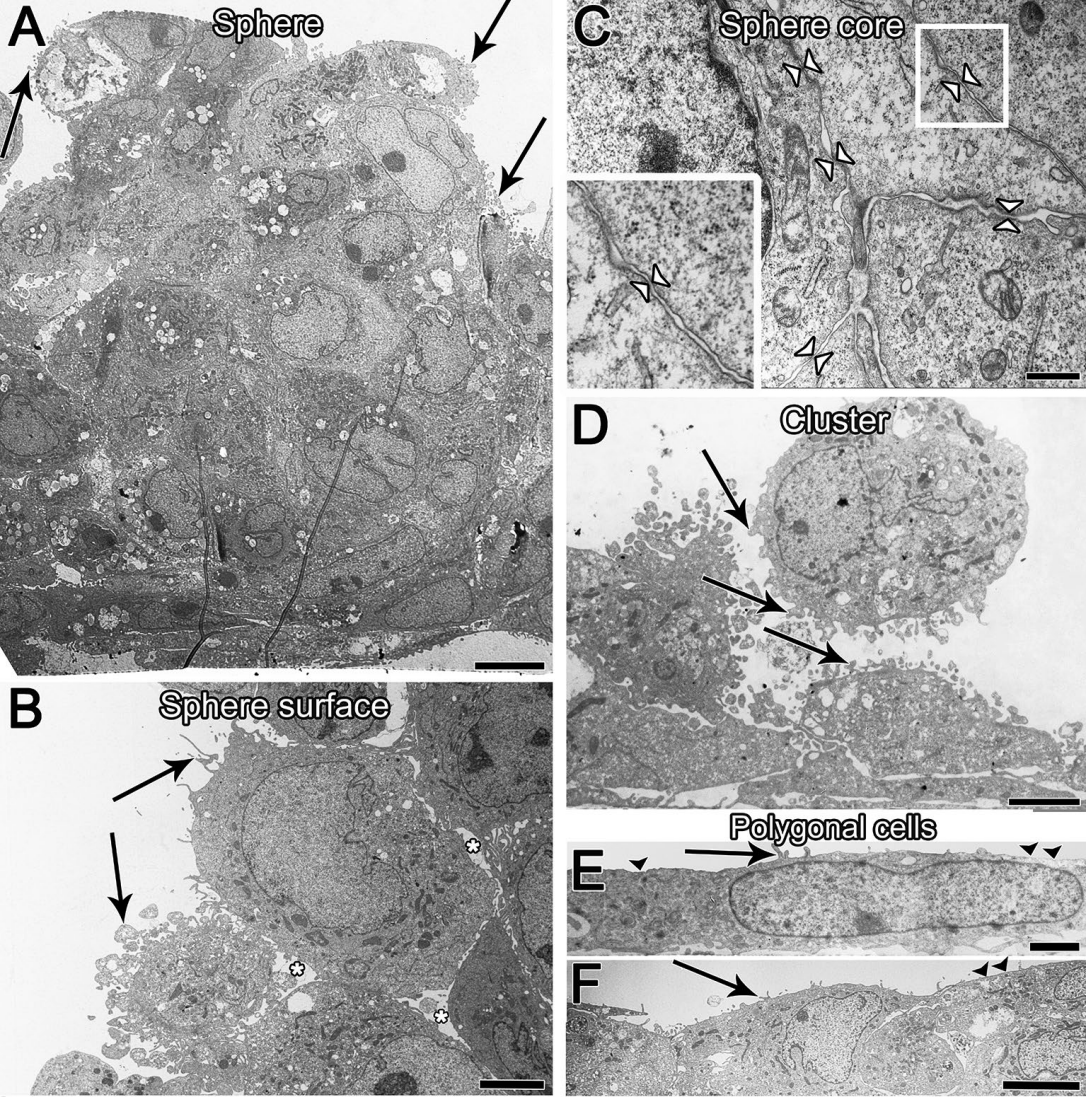
The ultrastructure of spheres, clusters, and polygonal cells observed in all FLO-1 models in vitro regardless of culture media. FLO-1 cells forming spheres are round or oval (**A**). The surface of cells located on the periphery of the spheres is shaped in round protrusions or microvilli or both (arrows, **A**,**B**) and cells are often loosely attached (**B**, asterisks indicate large intercellular spaces). Conversely, cells located inside the spheres have a relatively smooth surface and are more tightly connected (**C**, arrowheads indicate cell contacts). The cell surface of FLO-1 cells forming clusters is usually shaped in protrusions and microvilli (arrows, **D**). Cells are loosely attached (**D**). Cells forming a monolayer or bilayer are usually elongated and flattened, often with elongated ellipsoidal nuclei (**E**,F). Their surface is relatively smooth (arrowheads, **E**, **F**), or shaped in microvilli (arrows, **E**,**F**). Large inset framed with white lines in image C is 100% enlarged image of corresponding small white framed inset. Scale bars, 10 µm (**A**), 6 µm (**B**), 4 µm (**D**), 2 µm (**E**), 0.6 µm (**C**,**F**).

**Figure 6:**
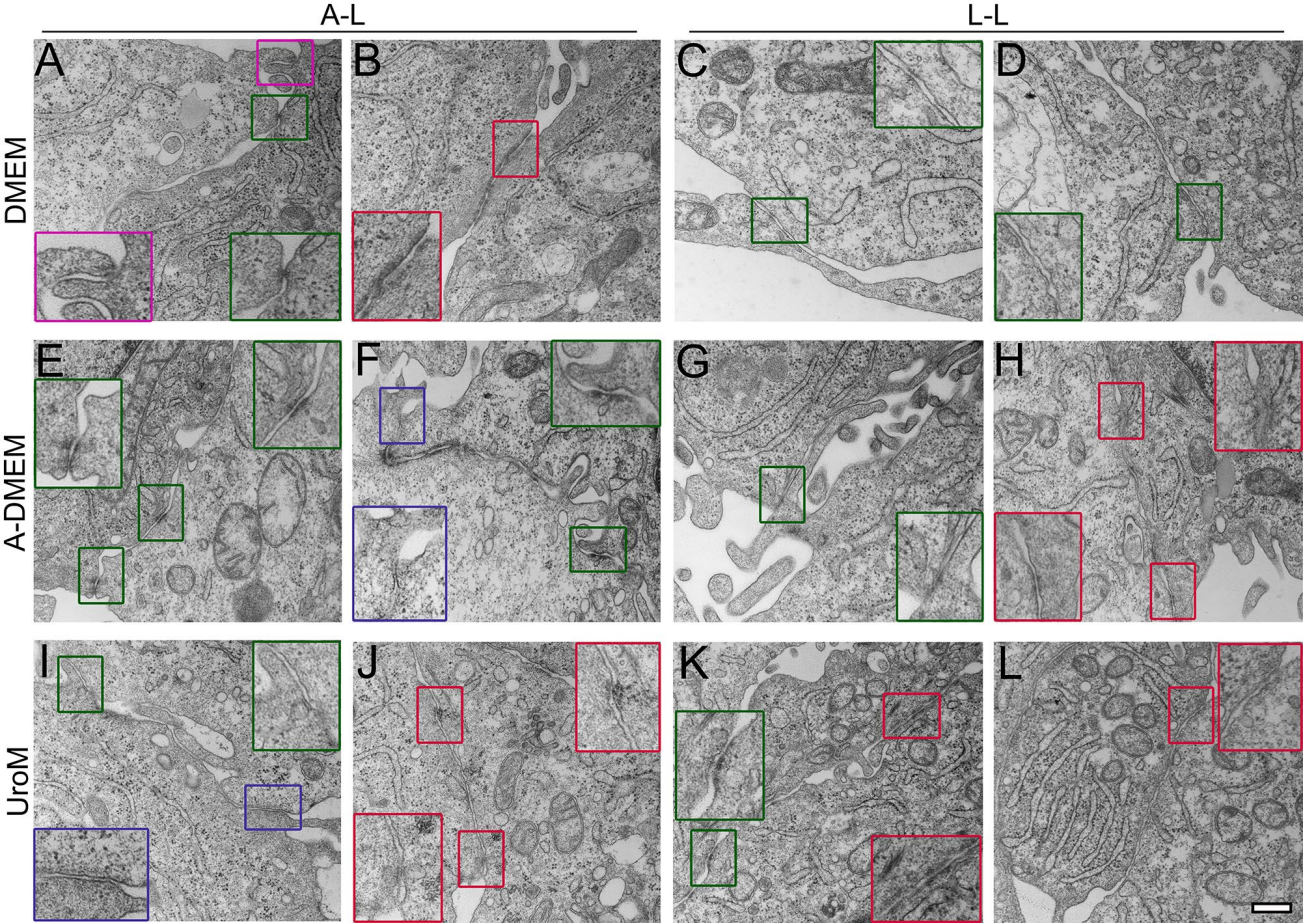
Ultrastructure of FLO-1 cells cultured on porous membranes and maintained in DMEM, A-DMEM and UroM for 4 wks at the A–L and L–L interfaces, analyzed with high magnification TEM. Cells are connected by protrusions (purple inset, **A**), anchoring junctions (green insets, **A**,**C**,**D**,**E**–**G**,**I**,**K**), adherent junctions and desmosomes (red insets, **B**,**H**,**J**–**L**). Cells maintained in A-DMEM and UroM are occasionally connected also via immature tight junctions (blue insets, **F**,**I**). Large insets framed with green/red/purple/blue lines are 100% enlarged images of corresponding small green/red/purple/blue framed insets. Scale bar, 400 nm.

TEM analysis confirmed cell shape heterogeneity in all FLO-1 cell models, i.e., maintained in different media types and interfaces (Fig. 5, Supplemental Fig. S8–10). Cells displayed relatively large, irregularly shaped nuclei and a small amount of cytoplasm. The surface was either smooth or shaped in irregular protrusions or microvilli, or both (Fig. 5, Supplemental Fig. S8–10). Cell spheres were composed of round or oval cells. The cell surface of cells located on the periphery of the spheres was very often shaped in round protrusions, microvilli, or both, although some superficial cells also exhibited smooth surface (Fig. 5A,B). Conversely, cells located inside the spheres had a relatively smooth surface (Fig. 5A,C). Furthermore, cells inside the spheres were frequently more tightly attached via anchoring and adherent junctions and desmosomes (Figs. 5C, 6, Supplemental Fig. S4), while cells on the periphery of spheres with microvilli and protrusion were more loosely attached to other cells via cell protrusions and anchoring junctions (Figs. 5B, 6, Supplemental Fig. S4). Small cell clusters exhibited similar ultrastructural properties as the spheres (Fig. 5D, Supplemental Fig. S8–10).

Polygonal cells forming a monolayer or bilayer very often had a relatively smooth surface with few microvilli (Fig. 5E,F). They were also more tightly attached to neighboring cells via adherent junctions and desmosomes or, less frequently, via tight junctions (Fig. 6, Supplemental Fig. S4). The medium type also influenced the development of cell junctions in FLO-1 models. Namely, FLO-1 cells maintained in A-DMEM and UroM were more often tightly connected to neighboring cells by adherent junctions and desmosomes, and even with individual tight junctions, than cells maintained in DMEM (Fig. 6, Supplemental Fig. S4). Except for the increased number of vacuolar structures mainly in the cells in spheres and clusters, no other peculiarities in the intracellular ultrastructure were observed (Fig. 5.).

### The effect of culture medium type on cell junction protein expression and TER of air–liquid and liquid–liquid interfaces FLO-1 models

Since FLO-1 models maintained in A-DMEM and UroM (both containing a lower serum concentration than DMEM medium) have similar ultrastructural properties, in the following experiments, the cells were maintained only in A-DMEM and compared with cells maintained in DMEM.

**Figure 7:**
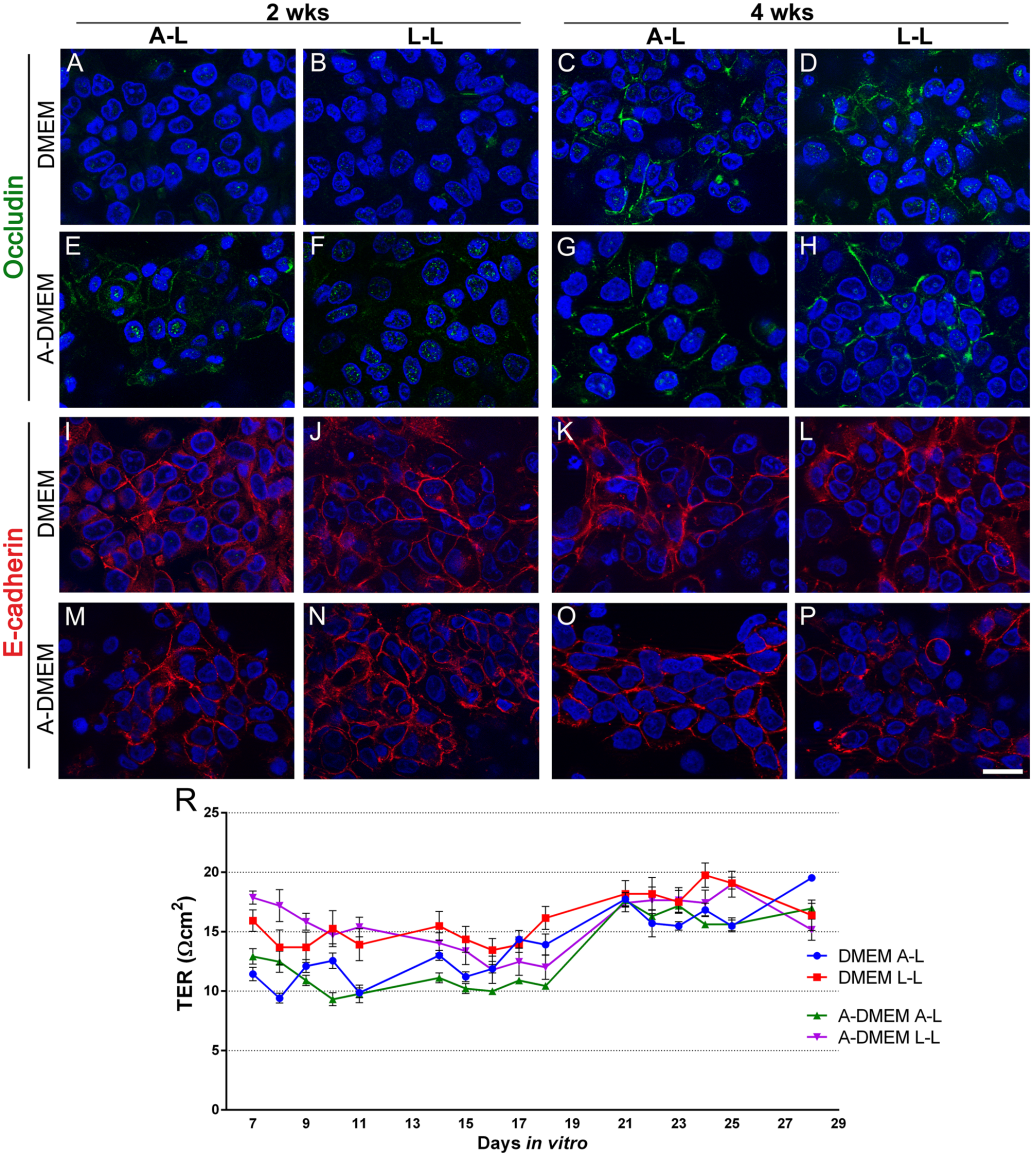
Occludin and E-cadherin expression and TER measurements in FLO-1 models. The cells grew on porous membranes in DMEM or A-DMEM culture media at the A–L and L–L interfaces for 2 or 4 wks. (**A**–**P**) Immunofluorescence labelling of occludin (**A**–**H**) and E-cadherin (**I**–**P**). Nuclei are stained blue. Occludin expression is higher in FLO-1 cells maintained in culture for 4 wks compared to 2 wks and when maintained in A-DMEM medium. All FLO-1 models exhibited E-cadherin immunoreactivity. Scale bar, 20 µm. (R) TER of different FLO-1 models at the A–L or L–L interface. The TER measurements started when the cells reached confluence (i.e., 7 days after the seeding). TER was then measured for another 3 wks (i.e., until the 28th day in vitro). FLO-1 models at the L–L interface show higher TER than at the A–L interface, except on the last day of measurements. However, all FLO-1 models exhibit very low TER. TER was measured in 4 independent cell cultures per group.

The expression of junctional proteins in FLO-1 models was further investigated with immunofluorescent labeling. In all media types, regions of cultures with high and low occludin expression were found (Supplemental Fig. S5). Areas with high occludin expression, where occludin was localized at apicolateral borders of adjacent cells, consisted of polygonal FLO-1 cells forming a monolayer or bilayer (Fig. 7A–H). In cell spheres and clusters, however, occludin expression was weak, with the immunofluorescence signal diffusely distributed throughout the cells and not located at the apicolateral borders (Supplemental Fig. S5). Consistent with the TEM observations, FLO-1 models maintained in A-DMEM exhibited higher occludin expression levels than FLO-1 models maintained in DMEM. In both media types, the expression of occludin was higher at 4 wks than at 2 wks of culturing (Fig. 7A–H).

E-cadherin, the protein of adherent junctions, was expressed in all FLO-1 models regardless of interface or medium type (Fig. 7I–P). E-cadherin was localized as a continuous line at the apicolateral border of adjacent cells in spheres and clusters as well as in regions where the cells formed a monolayer or bilayer (Fig. 7I–P). Individual cells not connected to adjacent cells did not display E-cadherin immunoreactivity.

The TER of established FLO-1 models was very low, regardless of the interface and the type of culture medium. The TER measurements started when the cells reached confluence (i.e., 1 week after the seeding). At that time, the measured TER values ranged between 10 in 20 Ωcm^2^, with the highest values in FLO-1 cultures maintained in the A-DMEM medium at the L–L interface. The TER values then slowly decreased at the L–L interface over the next two wks and then increased again in the last wk (fourth) of measurements, while at the A–L interface the TER slowly increased over the next 3 wks (Fig. 7R). Throughout the course of the TER measurements, the models cultured at the L–L interface showed higher TER values compared to the A–L interface, except for the last day of measurements, when the A–L interface model showed the highest TER values (Fig. 7R). Nevertheless, all FLO-1 models exhibited very low TER values compared to normal esophageal epithelia, which exhibit TER between 280 and 1000 Ωcm^2^^2,55–57^.

### Differentiation potential of FLO-1 cells under optimized conditions

Specific cancer cell lines have the potency to differentiate under optimized culture conditions and can partially mimic the properties of particular epithelia in vivo^33–36^. In this study, we have determined that cell viability is increased, and the formation of tight junction enhanced when FLO-1 cells were maintained in A-DMEM (supplemented with 2.5% FBS) (Figs. 2, 6). Therefore, we performed further molecular, ultrastructural, and functional (barrier function) characterizations of FLO-1 models maintained in A-DMEM medium to evaluate their differentiation potential. Cells were maintained in vitro at the A–L and L–L interfaces for short-term period, i.e., 1 wk, since the longer time of culturing resulted in cell shedding (Fig. 2) and because the TER values are relatively high at 1 wk in culture (compared to other examined time points; Fig. 7R.

**Figure 8:**
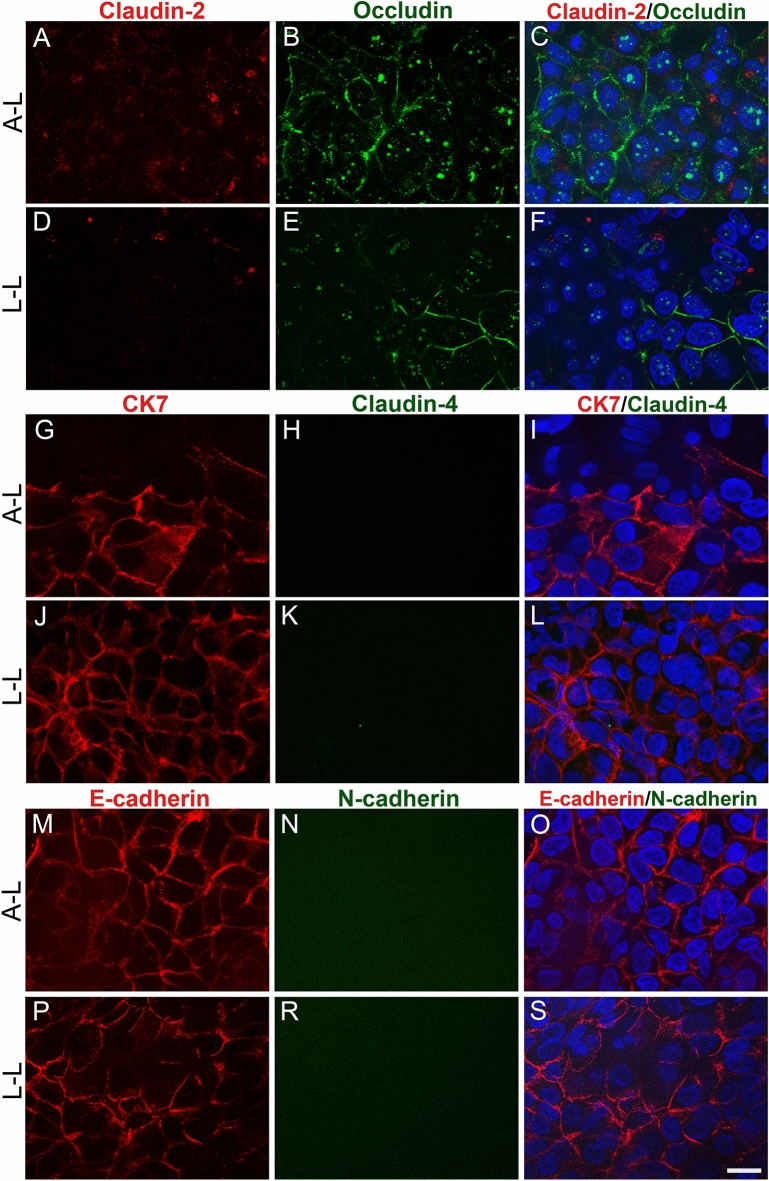
Expression of junctional proteins and cytokeratin-7 (CK-7) in short-term FLO-1 models. Cells were grown on porous membranes in A-DMEM medium at the A–L and L–L interfaces for 1 wk. Double immunofluorescence labelling of claudin-2 and occludin (**A**–**F**), cytokeratin 7 (CK7) and claudin-4 (**G**–**L**), and E-cadherin and N-cadherin (**M**–S). FLO-1 cells express occludin (green, **B**–**C**,**E**–**F**), CK7 (red, **G**,**I**,**J**,**L**) and E-cadherin (red, **M**,**O**,**P**,S) but not claudin-2 (**A**,**D**), claudin-4 (**H**,**K**) or N-cadherin (**N**,**R**). Nuclei are stained blue. Scale bars, 20 µm.

Short-term FLO-1 models were examined for cell junction proteins and cytokeratin 7 (CK-7) by double immunofluorescence labelling (Fig. 8). FLO-1 cells expressed the tight junction protein occludin, but not claudin-2, regardless of the growth interface (Fig. 8A–F). Occludin expression was higher when cells were grown at the A–L interface (Fig. 8B–C,E–F). It was expressed at apicolateral borders of adjacent cells in some culture areas. Cells growing in clusters or not connected to adjacent cells did not express occludin at the apicolateral borders; the immunosignal was dispersed throughout the cell. FLO-1 cells did not express claudin-4 (Fig. 8H–I,K–L), nor claudin-8 (Supplemental Fig. S6), regardless of the interface. Next, the expression of CK-7 was examined. CK-7 is a ductal marker expressed in the glandular mucosa in the esophagus^58^. CK-7 was expressed in the basal layers of FLO-1 models at the A–L and L–L interface, mainly localized near the cell periphery (Fig. 8G,I,J,L). Analysis of adherent junctional protein expression showed that both the A–L and L–L interfaces short-term FLO-1 models exhibited E-cadherin expression at the lateral borders (Fig. 8M,O,P,S). In contrast, cells did not express N-cadherin, regardless of their growth at the A–L or L–L interface (Fig. 8N–O,R–S).

**Figure 9:**
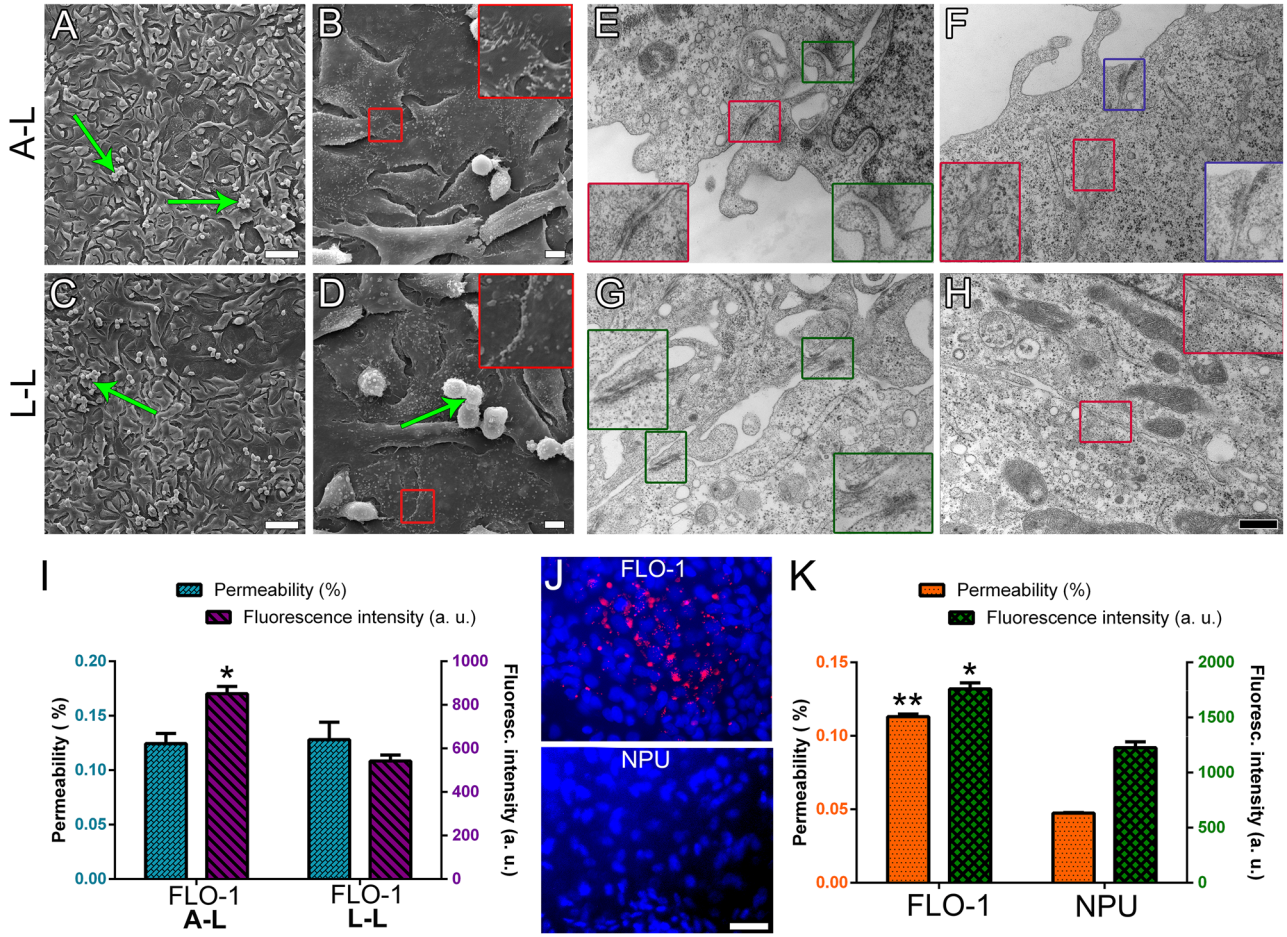
Ultrastructural and permeability analysis of short-term (1wk) FLO-1 in vitro models. Cells were grown on porous membranes in A-DMEM medium at the A–L and L–L interfaces for 1 wk. (**A–D**) SEM analysis shows polygonal FLO-1 cells and occasionally small cell clusters (green arrows) at both interfaces. Large insets framed with *red* lines are 200% enlarged images of the corresponding small *red-*framed insets. (**E–H**) TEM analysis shows that FLO-1 cells are connected *by* anchoring junctions (green insets), adherent junctions and desmosomes (red insets), and immature tight junctions (blue inset). Cells maintained at the A–L are more frequently attached via developing tight junctions than at the L–L interface. Large insets framed with *green*/*red/purple/blue* lines are 100% enlarged images of corresponding small green/*red/purple/blue* framed insets. (**I**,**K**) Quantification of permeability and fluorescence intensity of iNANO-RITC nanoparticles in FLO-1 and normal porcine urothelial (NPU) cells. The bars represent the average fluorescence values of iNANO-RITC nanoparticles in basal chamber expressed as % of fluorescence of nanoparticles added to the apical chamber (blue bars (**I**), orange bars (**K**)) and average fluorescence intensity in arbitrary units (a. u.) (purple bars (**I**) and green bars (**K**)). (**I**) Permeability of iNANO-RITC nanoparticles in FLO-1 cell grown at the A–L interface is similar to that at the L–L interface. The average fluorescence intensity is higher in FLO-1 models at the A–L interface (*p ≤ 0.05; un-paired Student’s t-test, n = 4). (**J**) Endocytosed iNANO-RITC nanoparticles (red) in FLO-1 and normal porcine urothelial (NPU) cells maintained at the L–L interface. (**K**) The permeability and fluorescence of endocytosed iNANO-RITC nanoparticles is significantly higher in FLO-1 cells compared to NPU cells (* ≤ 0.05; **p ≤ 0.00001; un-paired Student’s t-test, n = 12 for permeability measurements and n = 4 for fluorescence intensity measurements). Scale bars, 100 µm (**A**,**C**), 50 µm (**J**), 10 µm (**B**,**D**), and 400 nm (**E**–**H**).

Performing ultrastructural analysis of short-term FLO-1 models we have demonstrated that cells mostly grew in a monolayer, composed of polygonal cells with a smooth surface, at both interfaces (Fig. 9A–D). Cells were often connected to neighboring cells by adherent junctions and desmosome and immature tight junctions (Fig. 9E–H), which were more frequently found at the A–L interface. They also formed weak cell junction, i.e., anchoring junctions (Fig. 9E–G). Small clusters of round/oval FLO-1 cells with rough surface were also observed, but were less frequent compared to long-term (3–4 wks) FLO-1 models (Fig. 9A–D).

Further, cells grown in A-DMEM at the A–L or L–L interface were incubated with fluorescent iNANO-RITC nanoparticles. The measured permeability and fluorescence of endocytosed iNANO-RITC nanoparticles of FLO-1 models were then compared to highly differentiated in vitro model of the normal urothelium, which expresses tight junctions, has a high TER and forms the permeability barrier ^40,41,59–61^. The interface did not play a significant role in iNANO-RITC nanoparticles permeability in the short-term 1wk FLO-1 models (Fig. 9I). However, the measured fluorescence intensity of endocytosed nanoparticles in cells revealed a higher amount of iNANO-RITC nanoparticles in FLO-1 cells maintained at the A–L interface (Fig. 9I). In comparison to urothelial model, FLO-1 cells exhibited approximately 60% higher permeability (58 ± 3.6%, Fig. 9J–K) and 30% higher fluorescence intensity (30.3 ± 4.5%, Fig. 9J–K) of endocytosed iNANO-RITC nanoparticles, indicating low barrier integrity of FLO-1 models. Taken together, FLO-1 cells poorly mimic the properties of normal esophageal epithelium.

### Permeability of zero permeability markers and model drugs

The permeability of several zero permeability markers and model drugs was assessed in short-term (1 wk) FLO-1 cell models grown at the L–L interface, due to the cell shedding with prolonged culturing (Fig. 2) and higher TER values obtained in comparison with the A–L model (Fig. 7R), respectively.

**Figure 10:**
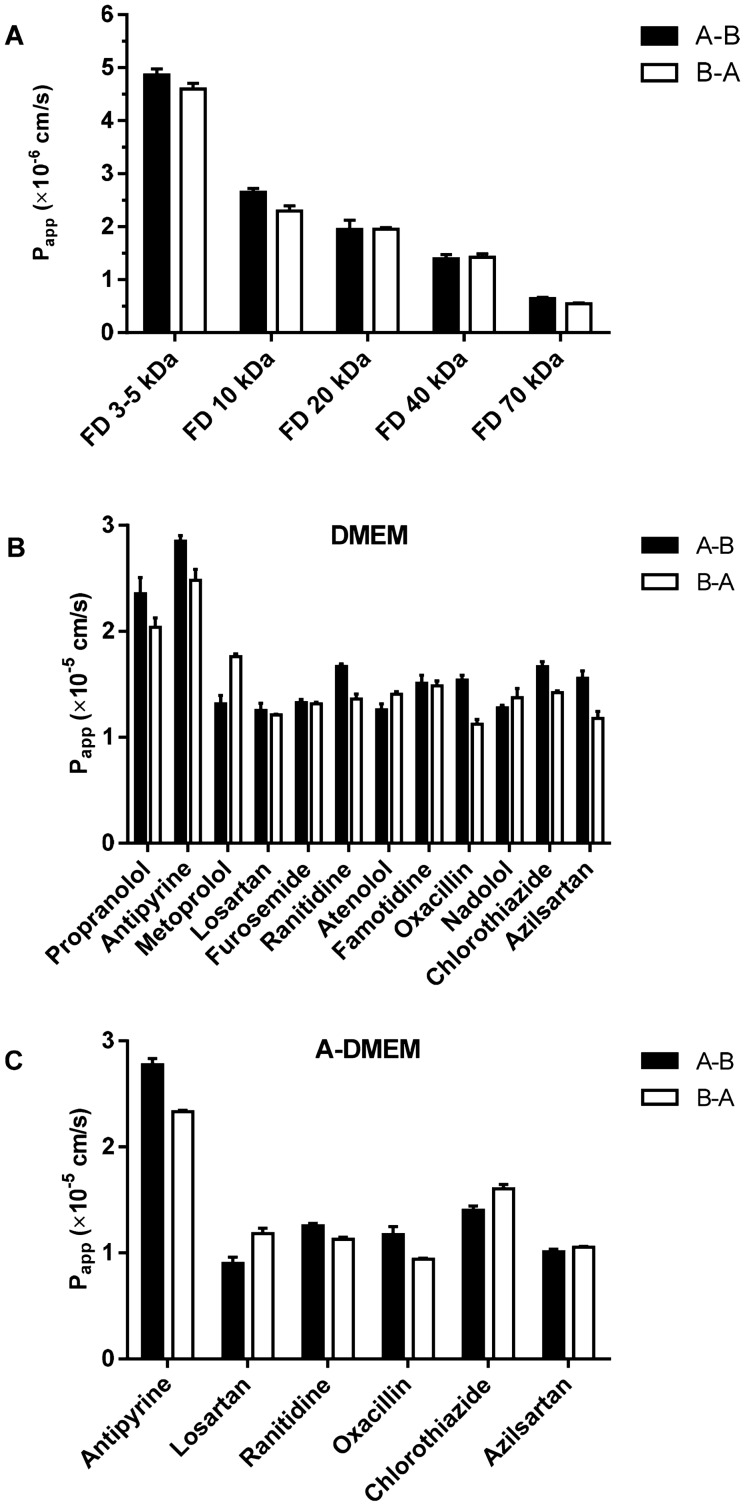
Average apparent permeability coefficients (P_app_s) in A-B and in B-A direction of: (**A**) zero permeability paracellular markers FITC-dextrans (FDs) with different MW, determined in the short-term 1wk FLO-1 model cultured in DMEM; (**B**) model drugs in FLO-1 cell model cultured in DMEM for 1 wk and; (**C**) model drugs FLO-1 cell model cultured in A-DMEM for 1 wk. N ≥ 3.

The apparent permeability coefficients (P_app_s) for the dextrans with different MW are shown in Fig. 10A. The lowest and the highest P_app_ values in FLO-1 models cultured in DMEM were obtained for the FD 70 kDa and FD 3–5 kDa, respectively, which is in line with their MW. The P_app_s for all the tested FDs, except for FD 70 kDa, are > 1.0 × 10^−6^ cm/s, indicating leakiness of the FLO-1 cell model.

Taking into account the recommendations for demonstrating the suitability of in vitro permeability methods for drug permeability classification from the recent ICH and FDA guidelines^62,63^, the permeability of several model drugs from the low, moderate and high permeability categories was assessed across the FLO-1 cells cultured in DMEM or A-DMEM (Fig. 10B–C). The highly permeable drugs permeate across the cell barrier by passive diffusion, while some of the low and moderately permeable drugs utilize the paracellular pathway or are known to be subject of active transport mechanisms. The obtained P_app_ values for the short-term FLO-1 models in the DMEM and in A-DMEM are shown in Supplemental Table 1 and in Fig. 10B and C, respectively. The determined efflux ratios for all tested compounds were close to 1 (0.72–1.33), suggesting that passive diffusion, but not active (efflux) transport, plays the primary role in the permeability of compounds through FLO-1 epithelium.

In the FLO-1 models cultured in DMEM, only a 1.5-fold difference was observed between the obtained P_app_ values for the highly permeable compounds propranolol and antipyrine and the P_app_ values of metoprolol, and the low and moderately permeable drugs. Metoprolol is suggested to be suitable permeability reference standard in intestinal permeability models, having permeability in the proximity of the low/high Biopharmaceutics Classification System (BCS) permeability class boundary^64,65^.

The P_app_ values for the low and moderately permeable model drugs determined in the FLO-1 model cultured in A-DMEM are significantly lower (*p* < 0.05) than the respective P_app_ values in the model cultured in DMEM. This is in agreement with the higher expression of tight junction proteins when A-DMEM is used as a cell culture medium (Figs. 6, 7A–H). The P_app_ value for highly permeable drug antipyrine is 2–threefold higher than values for the low and moderately permeable model drugs.

The P_app_ values for the low and moderately permeable drugs obtained in both cell models are of the same order of magnitude (× 10^−5^ cm/s) as the P_app_ values for the highly permeable drugs. This indicates the FLO-1 model is very leaky, which is substantiated by the measured low TER values (< 20 Ωcm^2^, Fig. 7R). The permeability of the tested model drugs was shown to be from 1.2-fold to 3.4-fold lower than the respective permeability across the PET membranes without cells. Thus, these results do not support the possibility of utilizing the FLO-1 cell line as a model of the esophageal epithelial barrier for drug permeability studies.

### FLO-1 model in submerged conditions

**Figure 11:**
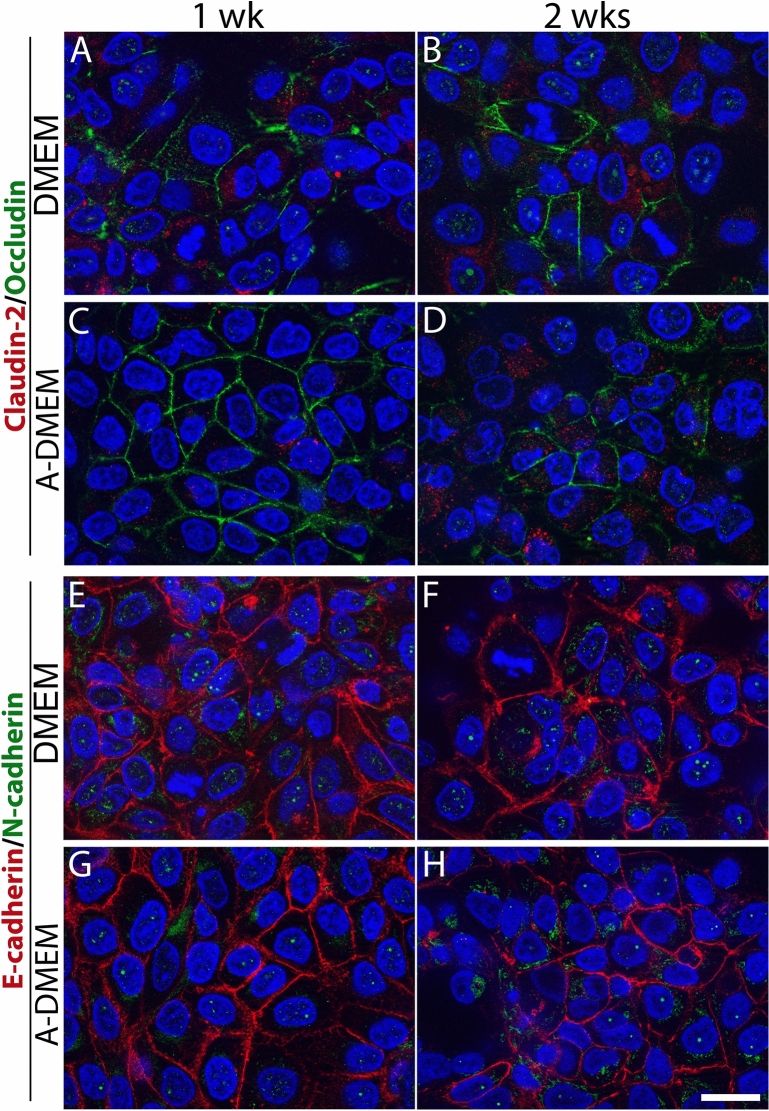
Expression of junctional proteins in submerged FLO-1 models. FLO-1 cells were maintained in DMEM or A-DMEM medium for 1 and 2 wks. Double immunofluorescence labelling of claudin-2 and occludin (**A**–**D**) and E-cadherin and N-cadherin (**E**–**H**). FLO-1 cells express occludin (green, **A**–**D**), and E-cadherin (red, **E**–**H**) in areas with polygonal cells, but claudin-2 (red, **A**–**D**) and N-cadherin (green, **E**–**H**) are only weakly expressed. Occludin expression is higher in cells maintained in A-DMEM and after 1 wk in culture. Scale bar, 20 µm.

In the last part of the study FLO-1 models were maintained in submerged conditions (seeded onto cell culture dishes) for 1 or 2 wks in DMEM or A-DMEM and then examined for junctional protein expression and ultrastructure. Like FLO-1 cells grown on porous membranes at the L–L interface, cells formed regions of polygonal-shaped cells and areas with cells growing in multi-layered (3–5 layers) clusters (Fig. 11A–D and Supplemental Fig. S7). Double immunofluorescence labelling revealed that FLO-1 cells expressed the tight junctional protein occludin in culture areas with polygonal cells, while claudin-2 was not expressed, regardless of the medium type. However, occludin expression was higher when cells were grown in A-DMEM (Fig. 11A–D), which is in agreement with the occludin expression in FLO-1 models grown on porous membranes (Figs. 6, 7). Occludin was expressed at apicolateral borders of adjacent cells in regions with polygonal FLO-1 cells forming a monolayer/bilayer (Fig. 11A–D, Supplemental Fig. S7). Cells growing in clusters or not connected to adjacent cells did not express occludin (Supplemental Fig. S7). The immunosignal was dispersed throughout the cells. In both media types, expression of occludin was higher at 1 wk compared to 2 wks of culturing (Fig. 11A–D, Supplemental Fig. S7). FLO-1 cells exhibited adherent protein E-cadherin at the apicolateral borders of adjacent cells in regions with polygonal cells as well as in clusters in both media (Fig. 11E–H). In contrast, low expression of N-cadherin was detected, regardless of their growth in DMEM or A-DMEM media (Fig. 11E–H).

**Figure 12:**
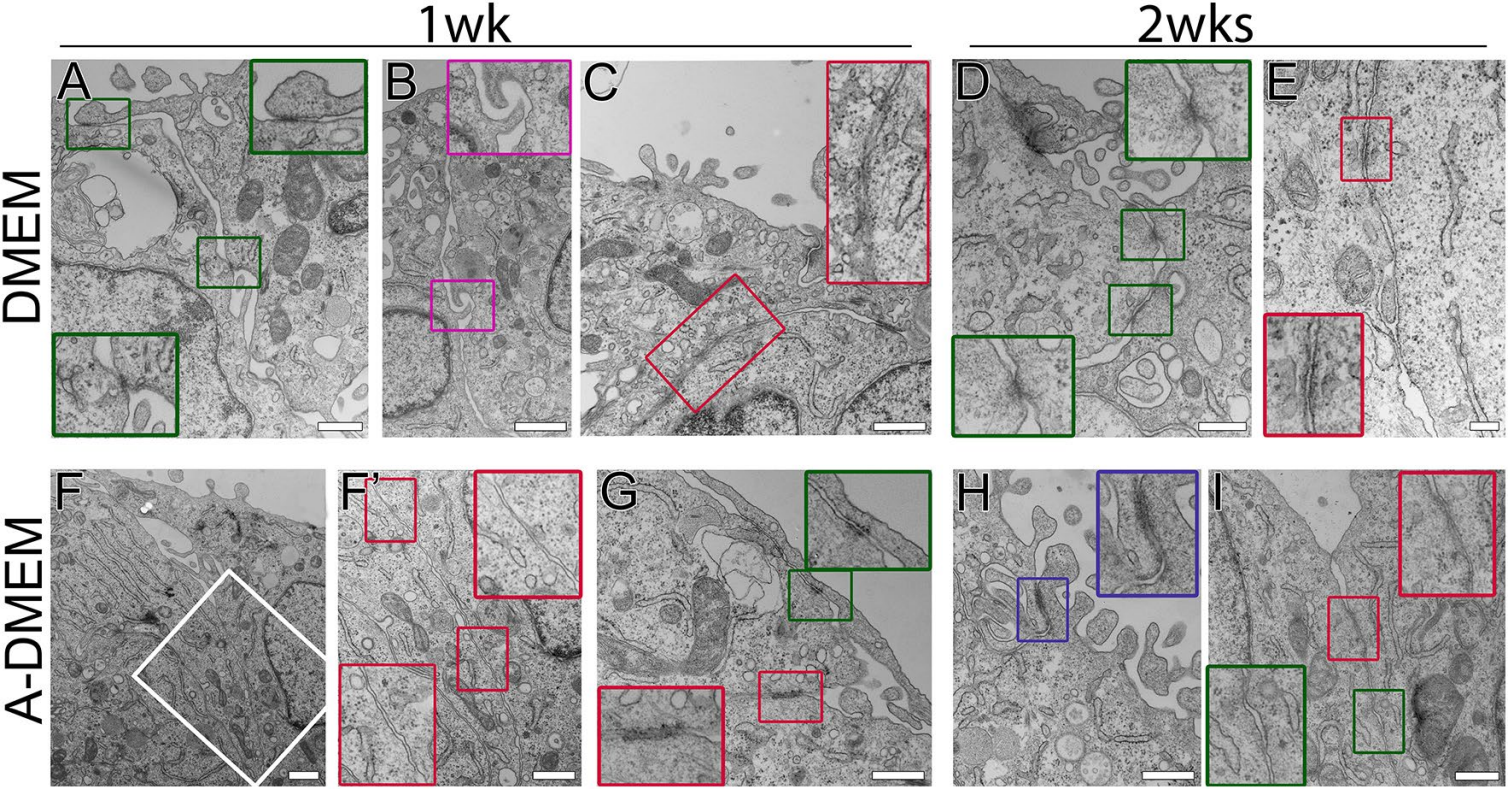
Ultrastructure of FLO-1 cells cultured in submerged conditions and maintained in DMEM or A-DMEM for 1 and 2 wks and analyzed with high magnification TEM. Cells are connected *by* protrusions (purple insets, **B**), anchoring junctions (green insets, **A**,**D**,**G**,**I**), adherent junctions and desmosomes (red insets, **C**,**E**,**F**’,**G**,**I**) and immature tight junctions (blue inset, **H**). Cells maintained in A-DMEM are more frequently attached via adherent and tight junctions as cells maintained in DMEM. Large insets framed with *green*/*red/purple/blue* lines are 100% enlarged images of corresponding small green/*red/purple/blue* framed insets. The white boxed area in image **F** is shown in the magnified view in image **F**’. Scale bar, 1 µm (**B**,**F**) 600 nm (**A**,**C**,**F**’,**G**,**H**,**I**), 400 nm (**D**), 200 nm (**E**).

Ultrastructural analysis of submerged FLO-1 models with TEM substantiated morphology heterogeneity shown with immunolabeling of junctional proteins. Cells exhibited either polygonal morphology, usually forming mono- or bilayer, or round to oval morphology, usually forming cell clusters (Supplemental Fig. S8–10). Cells were connected by protrusions, anchoring junctions, adherent junctions, and desmosomes and occasionally with developing tight junctions (Fig. 12). Immature tight junctions were more frequently seen in the cells maintained in A-DMEM than in DMEM (Fig. 12), supporting higher occludin expression in the FLO-1 model maintained in A-DMEM. Tight and adherent junctions and desmosomes were usually found between polygonal cells and less frequently between adjacent cells in spheres. Polygonal cells often exhibited a relatively smooth surface, with few microvilli. Cells inside spheres were usually tightly attached with anchoring junctions, while cells on the periphery of spheres loosely connected to other cells via cell protrusions (Fig. 12, Supplemental Fig. S8–10). No other peculiarities in the intracellular ultrastructure were observed (Fig. 12).

## Discussion

The effect of culturing conditions on the EAC cell line FLO-1 has not yet been evaluated. Therefore, the aim of this study was to characterize this cell line further and evaluate the culture conditions for its optimization and standardization as an in vitro model for EAC.

Cancer cell lines have been widely used to study the signaling pathways of the disease, to define potential molecular markers, and for the development, screening, and characterization of cancer therapeutics. They have also been recognized by biomedical and pharmaceutical companies as a model for drug testing and translational studies^66^. However, every cell line must be characterized with respect to culture conditions and environment in order to optimally mimic the in vivo situation for the investigated process. The optimization of culture conditions thus increases the validity of the in vitro model^67^.

The representability of human cancer cell lines of the in vivo cancer cell population can be augmented by simulation of physiological conditions as closely as possible in contrast to adapting the culturing conditions for maximum cell proliferation^67^. For example, animal-derived serum has been used as a medium supplement for the in vitro propagation of many normal as well as cancer cell lines. However, most cells are not exposed to serum under physiological conditions^68^. Furthermore, serum supplementation of culture media has additional disadvantages. It is a known source of variability, which makes it difficult to standardize experimental protocols. Proteomic and metabolomic studies showed that serum consists of about 1800 proteins and more than 4000 metabolites. Serum composition display qualitative, quantitative, geographical and seasonal batch-to-batch variations, originating in genetic diversity of source herds, animal nutrition and the serum production process^69,70^. The use of serum-free media is, therefore, key to obtaining more accurate results with cancer cell line models, which can facilitate more precise data analysis and interpretation^71^. Moreover, the serum is a potential source of contamination^72–74^. FBS may contain adverse factors such as endotoxins, mycoplasmas, viral contaminants, or prion proteins^70^. As cancer cell lines can be used directly for the production of new biological medicinal products (cell-based anti-tumor therapies^68,75–77^), which must be carried out under safe and controlled conditions, the development of standardized animal-products-free cell culture conditions is a necessity.

In the majority of the studies, FLO-1 cells are maintained in growth medium supplemented with 10% serum (DMEM with 10% FBS^14–16,18,19^). We have successfully cultured FLO-1 cells with high viability using substantially lower serum concentration. Firstly, we have utilized A-DMEM medium, which is enhanced basal medium formulation of DMEM enriched with normal serum components, allowing the cell cultivation at reduced serum concentrations. FLO-1 cells were successfully cultured in A-DMEM medium supplemented with only 2.5% FBS (Figs. 1, 2, 3, Supplemental Fig. S1–2). Secondly, cell growth was tested in serum-free defined medium formulated for epithelial cells, UroM. It consisted of equal amounts of MCDB153 and A-DMEM medium, both allowing growth in low serum concentrations, supplemented by several cell growth-supporting factors such as insulin and hydrocortisone. After an initial cell expansion in the 2.5% FBS supplemented UroM in the first week of culturing, the cells were successfully maintained for additional 1–3 wks in the serum-free UroM (Figs. 1, 2, 3). Taken together, our study demonstrates that cancer cell line FLO-1 can be cultured in substantially lower serum concentrations, which is an important step towards standardization and quality assurance.

In this study, we have characterized the influence of different culturing conditions on the morphological, ultrastructural and functional properties of FLO-1 cells in vitro; i.e., different media types, culturing interfaces and culturing times. The A–L interface culturing of FLO-1 cells was examined, as air is present in the esophagus^26,27^ and compared to the L–L interface culturing. We have demonstrated that the culture interface significantly affects the architecture of FLO-1 cell culture (Fig. 3). The latter plays an important role in the prediction efficacy of in vitro tests of new cancer therapies. In particular, the cell culture architecture considerably influences the response to anti-cancer drugs in in vitro cell line models^78–80^. Although the in vitro drug testing depends primarily on the use of monolayered subconfluent cell lines, they poorly imitate the conditions in vivo. For a better simulation of in vivo microenvironment, 3D cell cultures are used in cancer studies, such as spherical cultures and 3D tumor spheroids^81,82^. Multi-layered cell cultures, formed by cancer cell lines, which can grow in several layers after they reach confluence, also exhibit many properties of solid tumors, such as drug distribution into the tumor tissue^29–32^. For example, Movia et al. (2018) have demonstrated that post-confluent multilayered human adenocarcinoma alveolar basal epithelial cells A549 maintained at the A–L interface exhibit increased resistance to investigated benchmarking chemotherapeutics compared to sub-confluent monolayer 2D culture^80^. The increased resistance was comparable to 3D hypoxic tumor spheroids.

We have shown in this study that post-confluent culturing of FLO-1 cells at the A–L interface results in the formation of multi-layered spheres of grape-like appearance, with rough-surface (microvilli and round protrusions) cells predominantly located on the periphery of the spheres and smooth-surface cells inside of the spheres (Figs. 3, 4, 5). Large spheres of FLO-1 cells (100–200 µm) were formed in all media types, but at the A–L interface only, when maintained in culture for 4 wks. Shorter time of culturing i.e., 1–2 wks at the A–L interface resulted in cell forming small clusters of round/oval cells, surrounded by polygonal flattened cells with a relatively smooth surface (Figs. 3, 4). Similarly, at the L–L interface, small clusters of cells were formatted (up to 10 cells, diameter 50 µm) among areas of polygonal flattened cells forming a monolayer or bilayer. Culturing time did not affect cell architecture at the L–L interface. In submerged conditions, FLO-1 cultures exhibited similar cell architecture as when maintained at the L–L interface (Fig. 12, Supplemental Fig. S8–10).

Morphology and ultrastructure of FLO-1 spheres at the A–L interface were very similar to sphere formations of other cancer cell lines, such as pancreatic ductal adenocarcinoma cells (PDAC)^83^. It has been demonstrated that the formation of cancer cell spheres can be induced by ultra-low-attachment surfaces, hanging-drop method and scaffold culturing^84–86^. Here, we have demonstrated that cancer sphere formation can also be induced by the A–L interface culturing. Furthermore, studies have shown that such multicellular three-dimensional spheres enable the growth and proliferation of cancer stem cells, which are defined as  a subpopulation (small proportion) of cancer cells that possess a high tumorigenic potential and are responsible for tumor initiation, growth, and metastasis^83,85–88^. They are resistant to cytotoxic chemotherapy and ionizing radiation and are believed to be responsible for tumor recurrence and are therefore an important therapeutic target^83,88–91^.

Field of EAC is underrepresented in 3D in vitro models and the most common EAC in vitro research models are two-dimensional (2D) cell line cultures. The latter have been used in many studies to evaluate the therapeutic efficacy of different agents and mechanisms of intrinsic drug resistance (reviewed in ^92^). However, they are faced with the problem of standardized protocols and conditions for establishing EAC cell lines^92^. Furthermore, cross-contamination of cell lines and their mistaken identities have been major dilemmas for esophageal cancer research^11^. On the other hand, only a handful of 3D EAC in vitro models can be found in the literature. These include organotypic cultures^93^, matrix-embedded 3D cultures^94^, hollow scaffold-based approaches^95^, tumor explants^96^, multicellular tumor spheroids^97^ and recently established EAC organoids^98,99^. Nevertheless, most of the 3D tumor models are capable of mimicking only certain aspects of a tumor environment and thus are not an accurate representation of the complexity and heterogeneity of tumors. 3D models also face inconsistencies in size which impact the reproducibility of the data. Next, the limitations of 3D models are also non-uniform cell attachment and lack of high-throughput methods for tumor model formation^100,101^. There is therefore an urgent need for a high-throughput, relatively inexpensive 3D cell model which is fast and easy to develop, with controllable physical and spatial parameters, that can consistently and accurately represent the tumor microenvironment and provide reliable and reproducible data when used for in vitro drug testing^100^. This is especially true for the field of EAC research, due to the lack of 3D EAC in vitro models.

Thus, the A–L interface effect on post-confluent FLO-1 cell culture architecture can be implemented when using FLO-1 cell line in tumor biology and anticancer drug studies as a reliable and straightforward approach with the potential to increase the prediction efficiency of the model.

While the culturing interface influenced the cell culture architecture, the type of culture medium affected the viability and expression of the junctional proteins. Namely, the number of viable cells and expression of the tight junction protein occludin were increased in FLO-1 models maintained in the A-DMEM and UroM (Figs. 2, 6, 7, 11). Ultrastructural TEM analysis confirmed that tight junctions are occasionally developing between neighboring cells of FLO-1 cultures in areas of flattened polygonal cells in A-DMEM and UroM medium, whereas tight junctions were rarely found in DMEM medium (Figs. 6, 12). Nevertheless, all FLO-1 models displayed very low TER values (less than 20 Ωcm^2^, Fig. 7), suggesting that regardless of the growth medium type and growth interface FLO-1 cells do not form a tight barrier, as demonstrated for several cancer cell lines under optimized culture conditions, such as Calu3, Caco-2, and TR146^33,34,37,38^. In comparison, normal esophageal epithelium, which has a barrier function to protect the underlying tissue from the harmful intraluminal contents, exhibits TER values between 280 and 1000 Ωcm^2^^2,55–57^. In accordance with low TER values, FLO-1 models displayed high permeability of all model drugs with low MW (< 500 Da) (Fig. 10) and low expression of claudin-4, required for the regulation of the paracellular permeability of epithelial cells, which is inversely related to claudin-4 expression^102^. However, the obtained P_app_ values in the FLO-1 model for the zero permeability markers (FDs) having high MW (3–70 kDa) indicate that this cell line, despite its high leakiness, can distinguish between different high MW compounds.

Since our study could not demonstrate ability of this cell line to differentiate between the high and low permeable model drugs with low MW, the FLO-1 cell line is not suitable for utilization in drug permeability assays, i.e., when testing the permeability of potential drug candidates or investigating the effect of different composition of drug formulations on drug permeability. Nevertheless, other possibilities for utilizing the FLO-1 model in anticancer drug studies remain an option, such as testing the cytotoxic potential of anticancer drugs and efficacy in esophageal cancer treatment^19^, bearing in mind the aforementioned A–L interface effect on post-confluent FLO-1 cell culture architecture. Recently, a new therapeutic concept for esophageal cancer has been presented, in which a chemopreventive drug is applied to the distal esophagus in the form of an aerosol under pressure, i.e., pressurized intraluminal aerosol chemotherapy (PILAC)^103^. Furthermore, the A–L interface in vitro model maintained on the semipermeable porous membranes allows for easy post-treatment ultrastructural analysis, exposure of semi-dry apical cell surfaces to investigational formulations, and collection of secretion products from basal compartments. A–L interface EAC in vitro models may therefore represent an important tool in drug screening for the evaluation of aerosol-based therapeutics in the future.

Considering the importance of cadherin expression in tumor growth^104^, we have shown in our study that independent of the medium type, FLO-1 models expressed the adherent junction protein E-cadherin, an epithelial marker. It was located at the lateral borders between adjacent cells, while the mesenchymal marker N-cadherin, a feature of cells with high motility, was expressed cytoplasmatically in a more diffuse way (Fig. 11). Our results are in agreement with Liu et al. (2016), who demonstrated E-cadherin and N-cadherin expression in FLO-1 cells. In cancer cells, external stimuli of different microenvironmental conditions within the tumor during growth and metastasis induce epithelial-to mesenchymal transition (EMT) or mesenchymal-to-epithelial transition (MET) during which expression of N-cadherin and E-cadherin are induced or diminished^14^. During EMT/MET, cells are in a state of high plasticity, which is suggested to play an important role in the metastatic dissemination of carcinoma^105–108^. Liu et al. (2016) have shown that FLO-1 cells undergo EMT and metastasize following subcutaneous injection in mice^14^.

In conclusion, FLO-1 cells represent an important tool in EAC research as verified, authentic and non-contaminated cell line, which is easy to grow and manipulate in vitro. In this study, we have shown that its use as an in vitro model of EAC can be enhanced by optimized culturing conditions. Firstly, low serum and serum-free conditions can be implemented to achieve higher data reproducibility and to increase the validity of in vitro FLO-1 model, as the serum supplement does not represent physiological conditions. Secondly, post-confluent and A–L interface culture should be considered when the cell line is used in drug testing due to their effect on 3D architecture, which considerably influences the response to anti-cancer drugs in vitro*.* The characterization of the FLO-1 in vitro model, optimization of culturing conditions and their standardization therefore represent an important step towards the improved utility of the existing model, providing insights into the molecular mechanisms of tumor growth and metastasis of EAC and for the development of drug therapies.

## Supplementary Information

Supplementary Information

## Data Availability

The datasets generated during and/or analysed during the current study are available from the corresponding author on reasonable request.

## Abbreviations

A–L: Air–liquid
EAC: Esophageal adenocarcinoma
UroM: Epithelial medium
L–L: Liquid–liquid
SE: Standard error
SEM: Scanning electron microscopy
TEM: Transmission electron microscopy
TER: Transepithelial resistance
2D: Two-dimensional
3D: Three-dimensional

## References

[1] Napier KJ, Scheerer M, Misra S (2014). Esophageal cancer: a review of epidemiology, pathogenesis, staging workup and treatment modalities. World J. Gastrointest. Oncol..

[2] Pohl H, Sirovich B, Welch HG (2010). Esophageal adenocarcinoma incidence: are we reaching the peak?. Cancer Epidemiol. Biomarkers Prev..

[3] Chow WH (1998). Body mass index and risk of adenocarcinomas of the esophagus and gastric cardia. J. Natl. Cancer Inst..

[4] Whiteman DC (2008). Combined effects of obesity, acid reflux and smoking on the risk of adenocarcinomas of the oesophagus. Gut.

[5] Rubenstein JH, Shaheen NJ (2015). Epidemiology, diagnosis, and management of esophageal adenocarcinoma. Gastroenterology.

[6] Yousefi, M. *et al.* Vol. *5* (7) 2504–2517 (*Biomedical Research and Therapy*, 2018).

[7] Contino G (2016). Whole-genome sequencing of nine esophageal adenocarcinoma cell lines. F1000Res.

[8] Dulak AM (2013). Exome and whole-genome sequencing of esophageal adenocarcinoma identifies recurrent driver events and mutational complexity. Nat. Genet..

[9] Weaver JMJ (2014). Ordering of mutations in preinvasive disease stages of esophageal carcinogenesis. Nat. Genet..

[10] Layke JC, Lopez PP (2006). Esophageal cancer: a review and update. Am. Fam. Phys..

[11] Boonstra JJ (2010). Verification and unmasking of widely used human esophageal adenocarcinoma cell lines. J. Natl. Cancer Inst..

[12] Katt ME, Placone AL, Wong AD, Xu ZS, Searson PC (2016). In vitro tumor models: advantages, disadvantages, variables, and selecting the right platform. Front Bioeng. Biotechnol..

[13] Doke SK, Dhawale SC (2015). Alternatives to animal testing: a review. Saudi Pharm. J..

[14] Liu DS (2016). Novel metastatic models of esophageal adenocarcinoma derived from FLO-1 cells highlight the importance of E-cadherin in cancer metastasis. Oncotarget.

[15] Adams O (2018). A specific expression profile of LC3B and p62 is associated with nonresponse to neoadjuvant chemotherapy in esophageal adenocarcinomas. PLoS ONE.

[16] Tong Z (2017). Antitumor effects of cyclin dependent kinase 9 inhibition in esophageal adenocarcinoma. Oncotarget.

[17] Mari L (2018). microRNA 125a regulates MHC-I expression on esophageal adenocarcinoma cells, associated with suppression of antitumor immune response and poor outcomes of patients. Gastroenterology.

[18] Kohtz PD (2019). Toll-like receptor-4 is a mediator of proliferation in esophageal adenocarcinoma. Ann. Thorac. Surg..

[19] Kilari RS (2018). The cytotoxicity and synergistic potential of aspirin and aspirin analogues towards oesophageal and colorectal cancer. Curr. Clin. Pharmacol..

[20] Coecke S (2005). Guidance on good cell culture practice. A report of the second ECVAM task force on good cell culture practice. Altern. Lab. Anim..

[21] Hartung T (2002). Good cell culture practice. ECVAM good cell culture practice task force report 1. Altern. Lab. Anim..

[22] van der Valk J (2010). Optimization of chemically defined cell culture media–replacing fetal bovine serum in mammalian in vitro methods. Toxicol. In Vitro.

[23] Elhofy A (2008). Essential pharmaceuticals.

[24] Kc K, Rothenberg ME, Sherrill JD (2015). In vitro model for studying esophageal epithelial differentiation and allergic inflammatory responses identifies keratin involvement in eosinophilic esophagitis. PLoS ONE.

[25] Wu L (2018). Filaggrin and tight junction proteins are crucial for IL-13-mediated esophageal barrier dysfunction. Am. J. Physiol. Gastrointest. Liver Physiol..

[26] Goldwin RL, Heitzman ER, Proto AV (1977). Computed tomography of the mediastinum. Normal anatomy and indications for the use of CT. Radiology.

[27] Schraufnagel DE, Michel JC, Sheppard TJ, Saffold PC, Kondos GT (2008). CT of the normal esophagus to define the normal air column and its extent and distribution. AJR Am. J. Roentgenol..

[28] Liao C (2020). RAD51 inhibitor reverses etoposide-induced genomic toxicity and instability in esophageal adenocarcinoma cells. Arch. Clin. Toxicol. (Middlet).

[29] Minchinton AI, Tannock IF (2006). Drug penetration in solid tumours. Nat. Rev. Cancer.

[30] Cowan DS, Hicks KO, Wilson WR (1996). Multicellular membranes as an in vitro model for extravascular diffusion in tumours. Br. J. Cancer Suppl..

[31] Tannock IF, Lee CM, Tunggal JK, Cowan DS, Egorin MJ (2002). Limited penetration of anticancer drugs through tumor tissue: a potential cause of resistance of solid tumors to chemotherapy. Clin. Cancer Res..

[32] Ribatti D (2017). A revisited concept: contact inhibition of growth. From cell biology to malignancy. Exp. Cell Res..

[33] Jarc T (2019). Demonstrating suitability of the Caco-2 cell model for BCS-based biowaiver according to the recent FDA and ICH harmonised guidelines. J. Pharm. Pharmacol..

[34] Kreft ME (2015). The characterization of the human cell line Calu-3 under different culture conditions and its use as an optimized in vitro model to investigate bronchial epithelial function. Eur. J. Pharm. Sci..

[35] Kreft ME (2015). The characterization of the human nasal epithelial cell line RPMI 2650 under different culture conditions and their optimization for an appropriate in vitro nasal model. Pharm. Res..

[36] Wu J (2017). Characterization of air-liquid interface culture of A549 alveolar epithelial cells. Braz. J. Med. Biol. Res..

[37] Srinivasan B (2015). TEER measurement techniques for in vitro barrier model systems. J. Lab. Autom..

[38] Bierbaumer L, Schwarze UY, Gruber R, Neuhaus W (2018). Cell culture models of oral mucosal barriers: a review with a focus on applications, culture conditions and barrier properties. Tissue Barriers.

[39] Kreft ME (2010). Golgi apparatus fragmentation as a mechanism responsible for uniform delivery of uroplakins to the apical plasma membrane of uroepithelial cells. Biol. Cell.

[40] Višnjar T, Kocbek P, Kreft ME (2012). Hyperplasia as a mechanism for rapid resealing urothelial injuries and maintaining high transepithelial resistance. Histochem. Cell Biol..

[41] Tratnjek L, Romih R, Kreft ME (2017). Differentiation-dependent rearrangements of actin filaments and microtubules hinder apical endocytosis in urothelial cells. Histochem. Cell Biol..

[42] Kreft ME, Sterle M, Jezernik K (2006). Distribution of junction- and differentiation-related proteins in urothelial cells at the leading edge of primary explant outgrowths. Histochem. Cell Biol..

[43] Kreft ME, Sterle M, Veranic P, Jezernik K (2005). Urothelial injuries and the early wound healing response: tight junctions and urothelial cytodifferentiation. Histochem. Cell Biol..

[44] Kreft ME, Romih R, Sterle M (2002). Antigenic and ultrastructural markers associated with urothelial cytodifferentiation in primary explant outgrowths of mouse bladder. Cell Biol. Int..

[45] Jerman UD, Kreft ME (2018). Reuse of bladder mucosa explants provides a long lasting source of urothelial cells for the establishment of differentiated urothelia. Histochem. Cell Biol..

[46] Tratnjek L, Kreft M, Kristan K, Kreft ME (2020). Ciliary beat frequency of in vitro human nasal epithelium measured with the simple high-speed microscopy is applicable for safety studies of nasal drug formulations. Toxicol. In Vitro.

[47] Tadic M, Kralj S, Jagodic M, Hanzel D, Makovec D (2014). Magnetic properties of novel superparamagnetic iron oxide nanoclusters and their peculiarity under annealing treatment. Appl. Surf. Sci..

[48] Zablotsky D, Kralj S, Kitenbergs G, Maiorov MM (2020). Relating magnetization, structure and rheology in ferrofluids with multi-core magnetic nanoparticles. J. Nonnewton. Fluid Mech..

[49] Kralj S (2012). Effect of surface charge on the cellular uptake of fluorescent magnetic nanoparticles. J. Nanopart. Res..

[50] Kralj S, Drofenik M, Makovec D (2011). Controlled surface functionalization of silica-coated magnetic nanoparticles with terminal amino and carboxyl groups. J. Nanopart. Res..

[51] Kralj S, Makovec D (2015). Magnetic assembly of superparamagnetic iron oxide nanoparticle clusters into nanochains and nanobundles. ACS Nano.

[52] Petropoulou A (2020). Multifunctional gas and pH fluorescent sensors based on cellulose acetate electrospun fibers decorated with rhodamine B-functionalised core-shell ferrous nanoparticles. Sci. Rep..

[53] Sibinovska N, Žakelj S, Kristan K (2019). Suitability of RPMI 2650 cell models for nasal drug permeability prediction. Eur. J. Pharm. Biopharm..

[54] Center for drug evaluation and research. *Application number: 200796Orig1s000 Pharmacology review(s).* https://www.accessdata.fda.gov/drugsatfda_docs/nda/2011/200796Orig1s000PharmR.pdf (2010).

[55] Tobey NA, Argote CM, Vanegas XC, Barlow W, Orlando RC (2007). Electrical parameters and ion species for active transport in human esophageal stratified squamous epithelium and Barrett's specialized columnar epithelium. Am. J. Physiol. Gastrointest. Liver Physiol..

[56] Björkman E, Casselbrant A, Lundberg S, Fändriks L (2012). In vitro assessment of epithelial electrical resistance in human esophageal and jejunal mucosae and in Caco-2 cell layers. Scand. J. Gastroenterol..

[57] Orlando RC (2010). The integrity of the esophageal mucosa. Balance between offensive and defensive mechanisms. Best. Pract. Res. Clin. Gastroenterol..

[58] von Furstenberg RJ (2017). Porcine esophageal submucosal gland culture model shows capacity for proliferation and differentiation. Cell Mol. Gastroenterol. Hepatol..

[59] Višnjar T, Kreft ME (2013). Air-liquid and liquid-liquid interfaces influence the formation of the urothelial permeability barrier in vitro. Vitro Cell Dev. Biol. Anim..

[60] Lojk J (2018). Increased endocytosis of magnetic nanoparticles into cancerous urothelial cells versus normal urothelial cells. Histochem. Cell Biol..

[61] Kostevšek N (2018). Hybrid FePt/SiO. Nanoscale.

[62] European Medicines Agency. *ICH guideline M9 on biopharmaceutics classification system based biowaivers Step 2b, EMA/CHMP/ICH/4 93213/2018*, https://www.ema.europa.eu/en/documents/scientific-guideline/ich-m9-biopharmaceutics-classification-system-based-biowaivers-step-2b-first-version_en.pdf (2018).

[63] FDA U.S. Department of Health and Human Services Center for Drug Evaluation and Research. *Waiver of In Vivo Bioavailability and Bioequivalence Studies for Immediate-Release Solid Oral Dosage Forms Based on a Biopharmaceutics Classification System. Guidance for Industry*, https://www.fda.gov/media/70963/download (2017).

[64] Larregieu CA, Benet LZ (2014). Distinguishing between the permeability relationships with absorption and metabolism to improve BCS and BDDCS predictions in early drug discovery. Mol. Pharm..

[65] Zur M, Gasparini M, Wolk O, Amidon GL, Dahan A (2014). The low/high BCS permeability class boundary: physicochemical comparison of metoprolol and labetalol. Mol. Pharm..

[66] Ferreira, D., Adega, F. & Chaves, R. in *Oncogenomics and Cancer Proteomics—Novel Approaches in Biomarkers Discovery and Therapeutic Targets in Cancer* (ed César López-Camarillo and Elena Aréchaga-Ocampo) (IntechOpen, 2013).

[67] van Staveren WC (2009). Human cancer cell lines: experimental models for cancer cells in situ? For cancer stem cells?. Biochim. Biophys. Acta.

[68] Stadler G (2007). Development of standardized cell culture conditions for tumor cells with potential clinical application. Cytotherapy.

[69] Karnieli O (2017). A consensus introduction to serum replacements and serum-free media for cellular therapies. Cytotherapy.

[70] Gstraunthaler G, Lindl T, van der Valk J (2013). A plea to reduce or replace fetal bovine serum in cell culture media. Cytotechnology.

[71] Baker M (2016). Reproducibility: respect your cells!. Nature.

[72] Eloit M (1999). Risks of virus transmission associated with animal sera or substitutes and methods of control. Dev. Biol. Stand..

[73] Shah G (1999). Why do we still use serum in the production of biopharmaceuticals?. Dev. Biol. Stand..

[74] Wessman SJ, Levings RL (1999). Benefits and risks due to animal serum used in cell culture production. Dev. Biol. Stand..

[75] Dillman RO (2005). Cancer vaccine potency: is there a dose/response relationship for patient-specific vaccines and clinical outcomes?. Cancer Biother. Radiopharm..

[76] Michael A (2005). Delayed disease progression after allogeneic cell vaccination in hormone-resistant prostate cancer and correlation with immunologic variables. Clin. Cancer Res..

[77] Zhou X (2005). Diverse CD8+ T-cell responses to renal cell carcinoma antigens in patients treated with an autologous granulocyte-macrophage colony-stimulating factor gene-transduced renal tumor cell vaccine. Cancer Res..

[78] Padrón JM (2000). The multilayered postconfluent cell culture as a model for drug screening. Crit. Rev. Oncol. Hematol..

[79] Pizao PE (1993). Cytotoxic effects of anticancer agents on subconfluent and multilayered postconfluent cultures. Eur. J. Cancer.

[80] Movia D, Bazou D, Volkov Y, Prina-Mello A (2018). Multilayered Cultures of NSCLC cells grown at the Air–Liquid Interface allow the efficacy testing of inhaled anti-cancer drugs. Sci. Rep..

[81] Sant S, Johnston PA (2017). The production of 3D tumor spheroids for cancer drug discovery. Drug Discov. Today Technol..

[82] Joseph, J. S., Malindisa, S. T. & Ntwasa, M. in *Cell Culture* (ed Radwa Ali Mehanna) (IntechOpen, 2018).

[83] Ishiwata T (2018). Electron microscopic analysis of different cell types in human pancreatic cancer spheres. Oncol. Lett..

[84] Bahmad HF (2018). Sphere-formation assay: three-dimensional. Front. Oncol..

[85] Morrison BJ, Steel JC, Morris JC (2012). Sphere culture of murine lung cancer cell lines are enriched with cancer initiating cells. PLoS ONE.

[86] Amaral RLF, Miranda M, Marcato PD, Swiech K (2017). Comparative analysis of 3D bladder tumor spheroids obtained by forced floating and hanging drop methods for drug screening. Front. Physiol..

[87] Reya T, Morrison SJ, Clarke MF, Weissman IL (2001). Stem cells, cancer, and cancer stem cells. Nature.

[88] Ishiwata T (2016). Cancer stem cells and epithelial-mesenchymal transition: novel therapeutic targets for cancer. Pathol. Int..

[89] Dean M, Fojo T, Bates S (2005). Tumour stem cells and drug resistance. Nat. Rev. Cancer.

[90] Donnenberg VS, Donnenberg AD (2005). Multiple drug resistance in cancer revisited: the cancer stem cell hypothesis. J. Clin. Pharmacol..

[91] Shibata M, Hoque MO (2019). Targeting cancer stem cells: a strategy for effective eradication of cancer. Cancers (Basel).

[92] Liu DS, Duong CP, Phillips WA, Clemons NJ (2016). Preclinical models of esophageal adenocarcinoma for drug development. Discov. Med..

[93] Underwood TJ (2010). A comparison of primary oesophageal squamous epithelial cells with HET-1A in organotypic culture. Biol. Cell.

[94] Andl CD, McCowan KM, Allison GL, Rustgi AK (2010). Cathepsin B is the driving force of esophageal cell invasion in a fibroblast-dependent manner. Neoplasia.

[95] Wang DH (2010). Aberrant epithelial-mesenchymal Hedgehog signaling characterizes Barrett's metaplasia. Gastroenterology.

[96] Seymour CB, Mothersill C, Cusack A, Hennessy TP (1988). The effect of radiation on the growth of normal and malignant human oesophageal explant cultures pre-treated with bleomycin. Br. J. Radiol..

[97] Zhao R, Quaroni L, Casson AG (2012). Identification and characterization of stemlike cells in human esophageal adenocarcinoma and normal epithelial cell lines. J. Thorac. Cardiovasc. Surg..

[98] Li X (2018). Organoid cultures recapitulate esophageal adenocarcinoma heterogeneity providing a model for clonality studies and precision therapeutics. Nat. Commun..

[99] Derouet MF (2020). Towards personalized induction therapy for esophageal adenocarcinoma: organoids derived from endoscopic biopsy recapitulate the pre-treatment tumor. Sci. Rep..

[100] Menon, J. U. 3D tumor models for cancer drug discovery: Current status and outlook. **2**, 1–2 (2018). https://oatext.com/3d-tumor-models-for-cancer-drug-discovery-current-status-and-outlook.php#Article_Info.

[101] Ricci C, Moroni L, Danti S (2013). Cancer tissue engineering—new perspectives in understanding the biology of solid tumours: a critical review. OA Tissue Eng..

[102] Cong X (2015). Claudin-4 is required for modulation of paracellular permeability by muscarinic acetylcholine receptor in epithelial cells. J. Cell Sci..

[103] Khalili-Harbi N (2016). Pressurized intraluminal aerosol chemotherapy with Dbait in the distal esophagus of swine. Endoscopy.

[104] Kaszak I (2020). Role of cadherins in cancer—a review. Int. J. Mol. Sci..

[105] Jolly MK (2019). Hybrid epithelial/mesenchymal phenotypes promote metastasis and therapy resistance across carcinomas. Pharmacol. Ther..

[106] Pastushenko I, Blanpain C (2019). EMT transition states during tumor progression and metastasis. Trends Cell Biol..

[107] Ishay-Ronen D, Christofori G (2019). Targeting cancer cell metastasis by converting cancer cells into fat. Cancer Res..

[108] Banyard J, Bielenberg DR (2015). The role of EMT and MET in cancer dissemination. Connect Tissue Res..

