# Supplementary material for: Standardization of esophageal adenocarcinoma in vitro model and its applicability for model drug testing
Source: Sci Rep. 2021 Mar 23;11:6664. doi: 10.1038/s41598-021-85530-w (PMC7988140; doi:10.1038/s41598-021-85530-w)
Supplement: Supplementary file 1 — Supplementary Information [file 41598_2021_85530_MOESM1_ESM.docx]

**SUPPLEMENTARY INFORMATION**

**Standardization of esophageal adenocarcinoma *in vitro* model and its applicability for model drug testing**

Larisa Tratnjek^a^, Nadica Sibinovska^b^, Slavko Kralj^c^, Darko Makovec^c^, Katja Kristan^d^*, Mateja Erdani Kreft^a^*

^a^University of Ljubljana, Faculty of Medicine, Institute of Cell Biology, Vrazov trg 2, 1000 Ljubljana, Slovenia

^b^University of Ljubljana, Faculty of Pharmacy, Chair of Biopharmaceutics and Pharmacokinetics, Aškerčeva c. 7, SI- 1000 Ljubljana, Slovenia

^c^Jožef Stefan Institute, Department for Materials Synthesis, Jamova 39, 1000 Ljubljana, Slovenia

^d^Lek Pharmaceuticals, d.d., Sandoz Development Center Slovenia, Verovškova 57, 1526 Ljubljana, Slovenia

*Corresponding authors: K. Kristan and M. E. Kreft

K. Kristan

Lek Pharmaceuticals, d.d., Sandoz Development Center Slovenia, Verovškova 57, 1526 Ljubljana, Slovenia

E-mail: katja.kristan@sandoz.com

Tel.: +386 1 580 34 61, Fax: +386 1 568 35 17

M. E. Kreft

Institute of Cell Biology, Faculty of Medicine, University of Ljubljana, Vrazov trg 2, SI-1000 Ljubljana, Slovenia

E-mail: mateja.erdani@mf.uni-lj.si

Tel.: +386 1 543 76 85, Fax: +386 1 543 7681

**Supplementary Figures:**

**
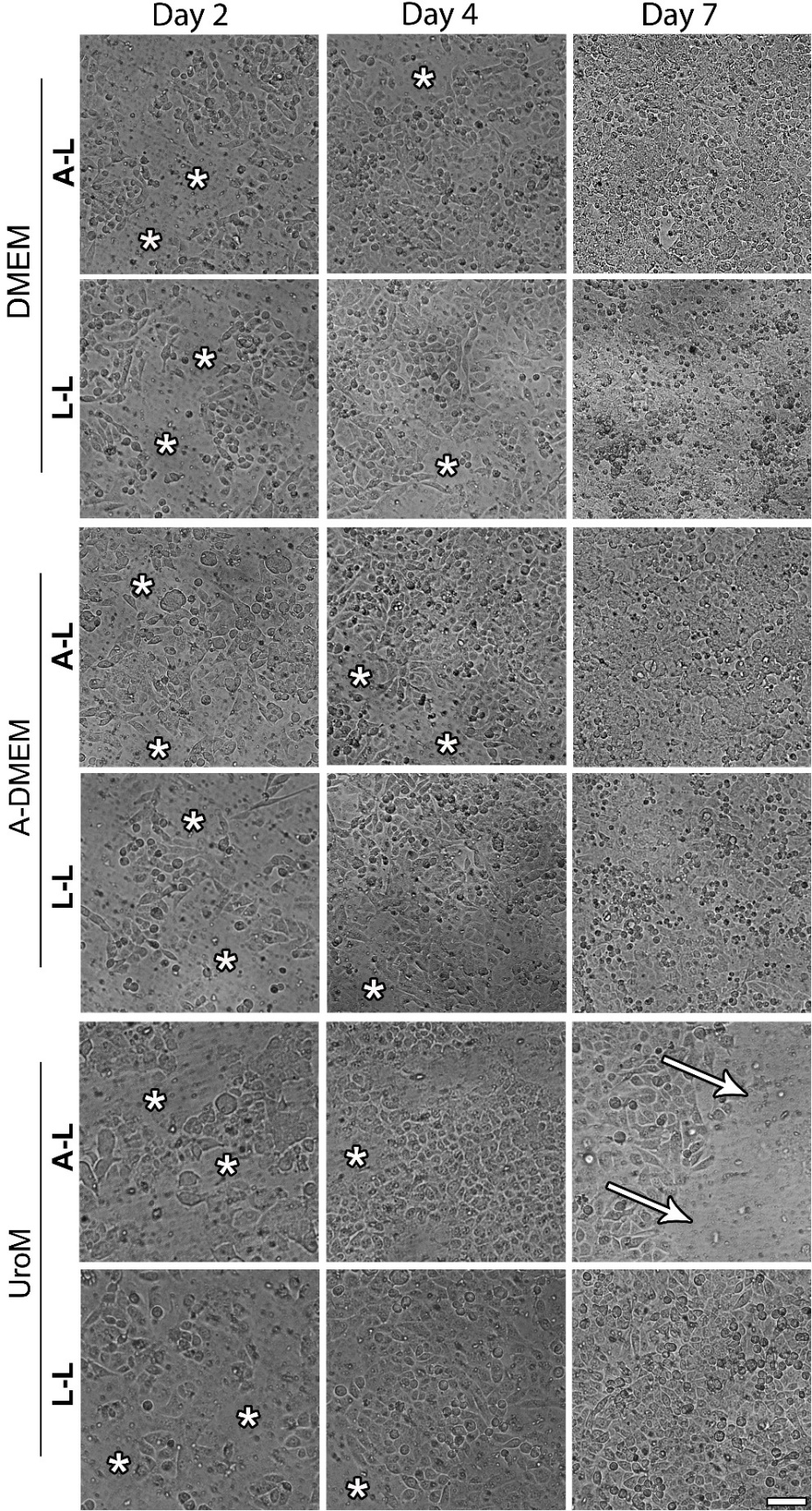
**

**Supplemental Figure S1**. FLO-1 cell proliferation in DMEM, A-DMEM and UroM culture media seeded with a density of 6×10^4^ cells/cm^2^ on polystyrene culture flasks on day 2, 4 and 7 after the seeding observed with phase-contrast microscopy. Asterisks indicate the areas of growth surface (porous membrane) not covered with cells. Cells reached confluence after 7 days, except for cell grown at the A-L interface in UroM, where cell shedding (white arrows) occurs even before cells reached confluence. Scale bar, 100 µm.


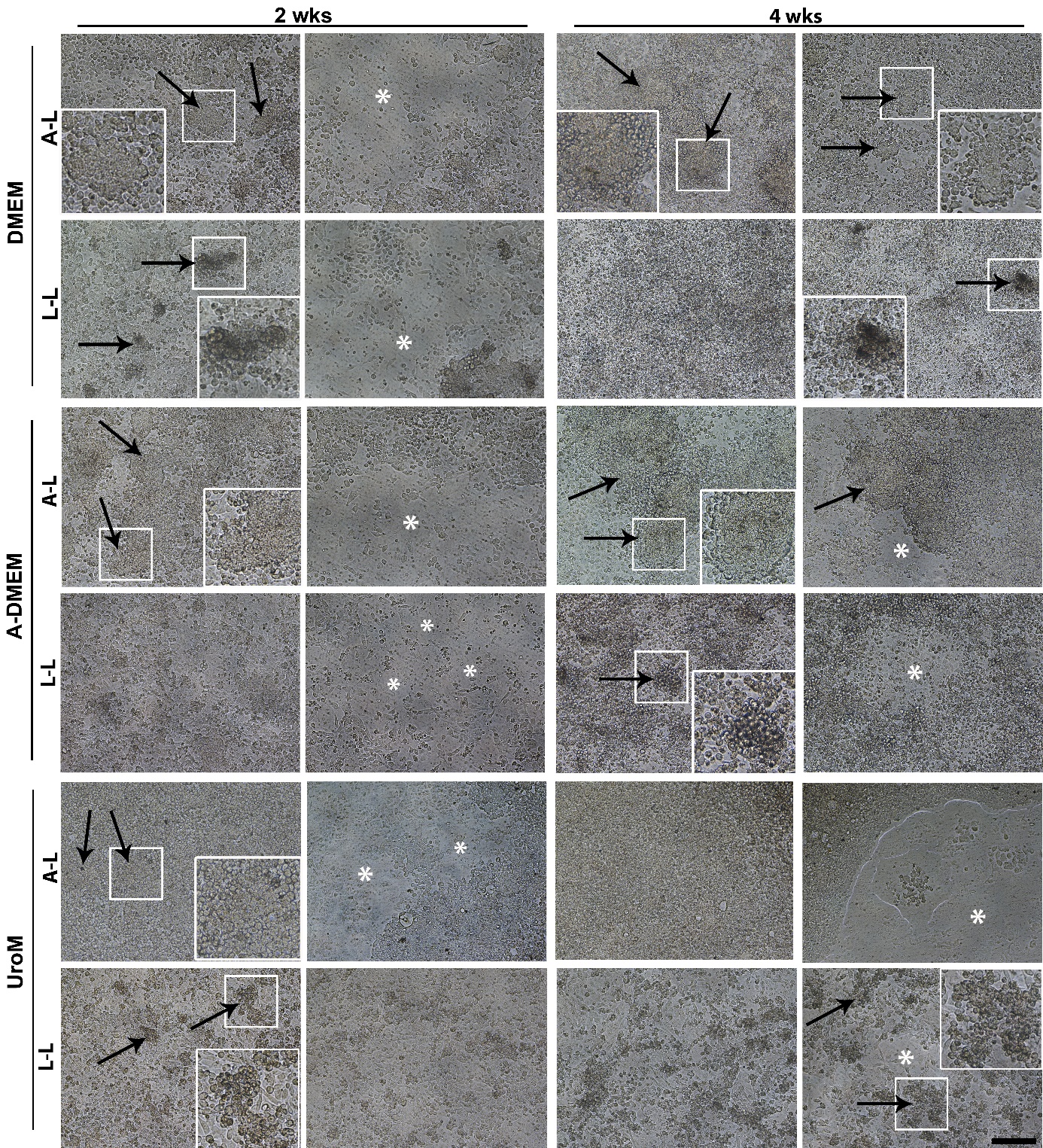


**Supplemental Figure S2**. FLO-1 cell proliferation in DMEM, A-DMEM and UroM culture media seeded with a density of 6×10^4^ cells/cm^2^ on polystyrene culture flasks on day 14 (2 wks) and 28 (4 wks) after the seeding observed with phase-contrast microscopy. Cells were maintained at the A-L or L-L interface. FLO-1 cells form spheres, pronounced especially at A-L interface (black arrows). Large insets framed with *white* lines are 100% enlarged images of corresponding small white framed insets. However, cell shedding (white asterisk) is observed in long-term culturing of FLO-1 cells, regardless of the culture media type. Cell shedding is less pronounced in FLO-1 cultures grown at the L-L interface in UroM. Scale bar, 100 µm.


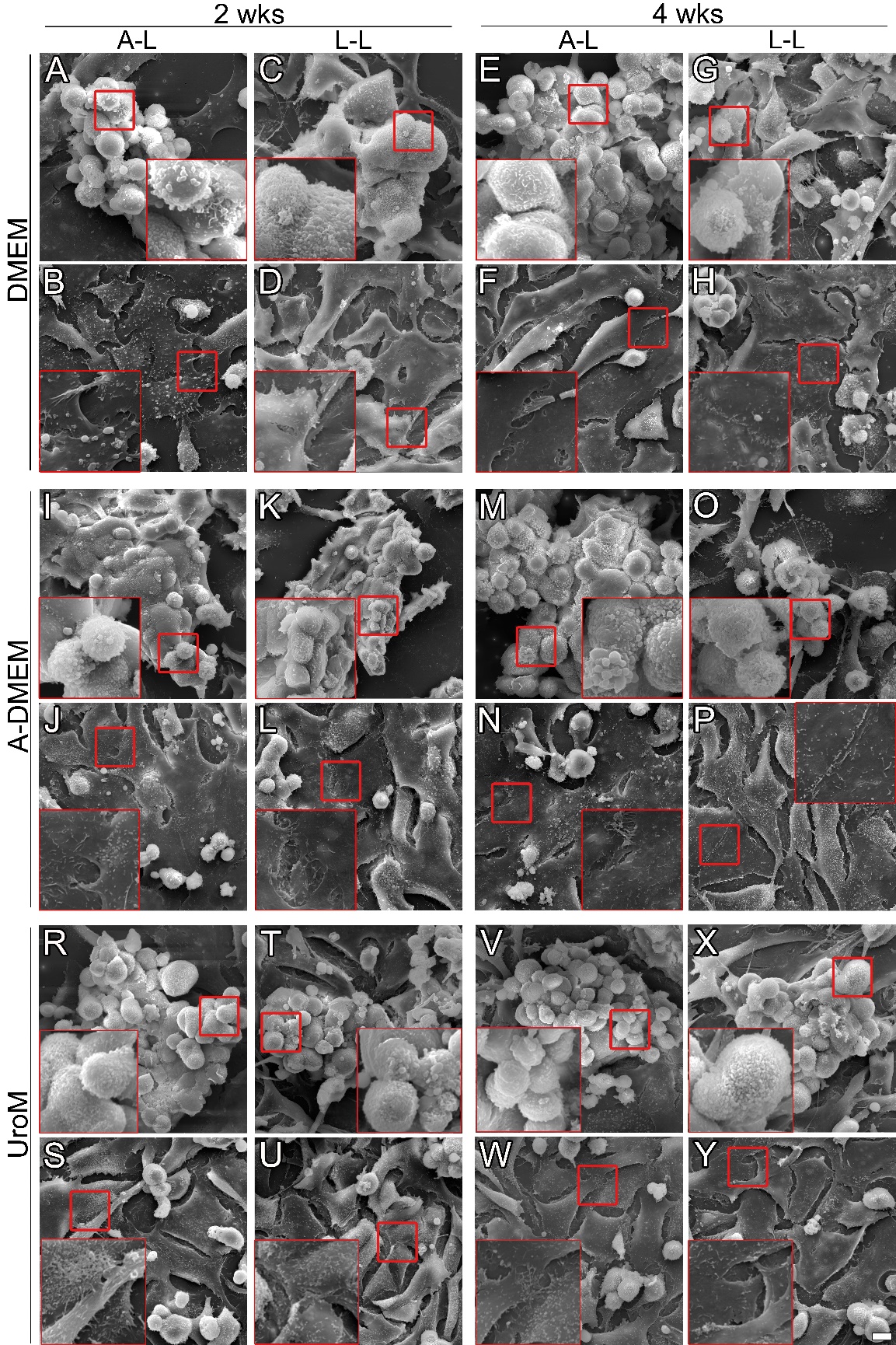


**Supplemental Figure S3.** FLO-1 cells cultured on porous membrane supports and maintained in DMEM, A-DMEM and UroM media for 2 and 4 wks at the A-L and L-L interface and analysed with high magnification SEM. In all media types, spheres and clusters and L-L interface (C, G, K, O T, X) respectively, consist of round to oval cells, which often have a rough surface (A, E, I, M, R, V). Spheres and clusters are surrounded by polygonal cells, which show a smoother surface (B, D, F, H, J, L, N, P, S, U, W, Y). Large insets framed with *red* lines are 150% enlarged images of corresponding small *red* framed insets. Scale bar, 10 µm


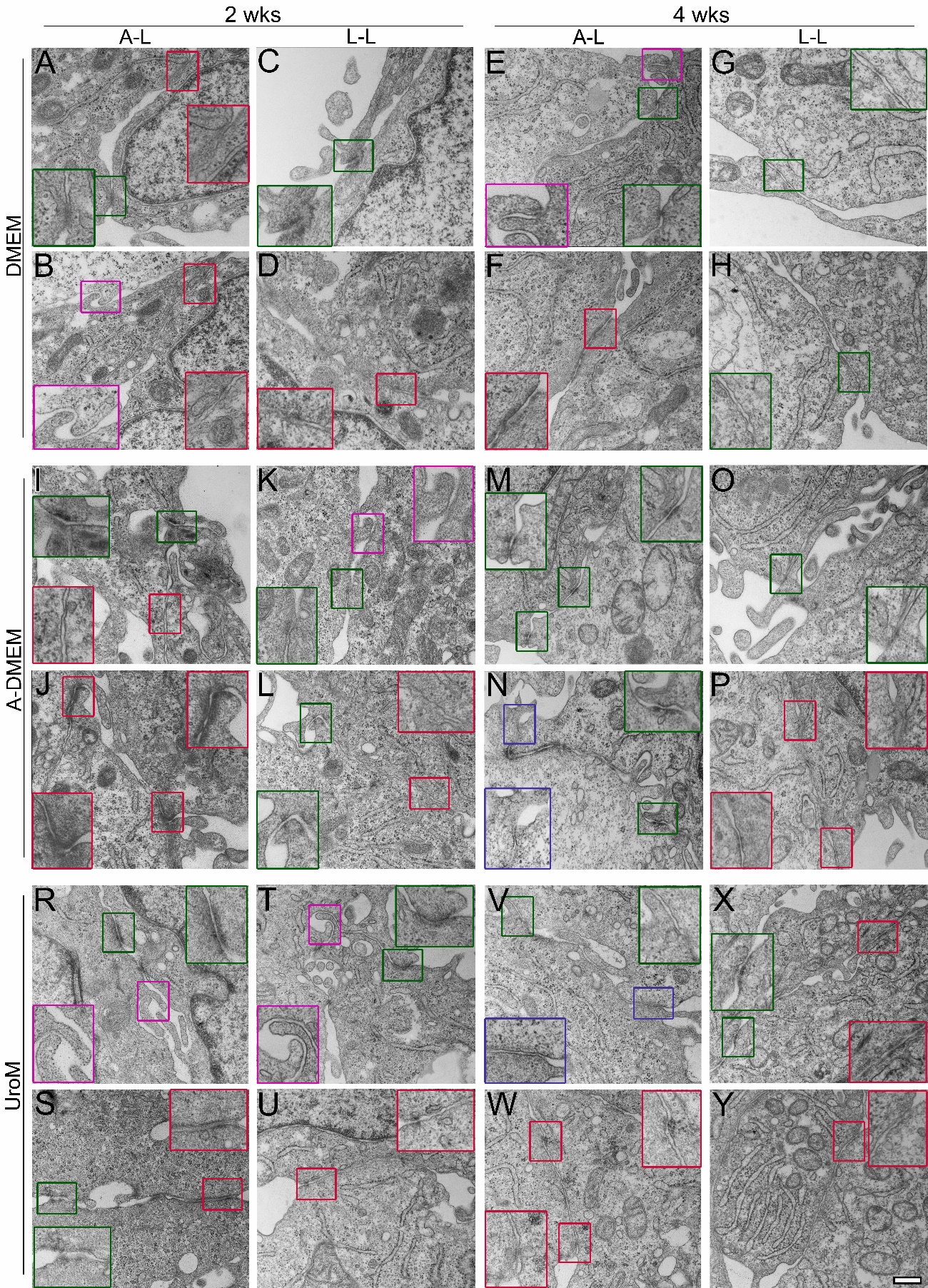


**Supplemental Figure S4.** Ultrastructure of FLO-1 cells cultured on porous membranes and maintained in DMEM, A-DMEM and UroM for 2 and 4 wks at the A-L and L-L interface, analysed with high magnification TEM. Cells are connected *by* protrusions (purple insets, B, E, K, R-T), anchoring junctions (green insets, A, C, E, G-H, I, K-O, R-T, V, X), adherent junctions and desmosomes (red insets, A-B, D, F, I-J, L, P, S, U, W, X-Y) and immature tight junctions (blue insets, N, V). Cells maintained in A-DMEM and UroM are occasionally connected also *via* tight junctions. Large insets framed with *green*/*red/purple/blue* lines are 100% enlarged images of corresponding small green/*red/purple/blue* framed insets. Scale bar, 400 nm.


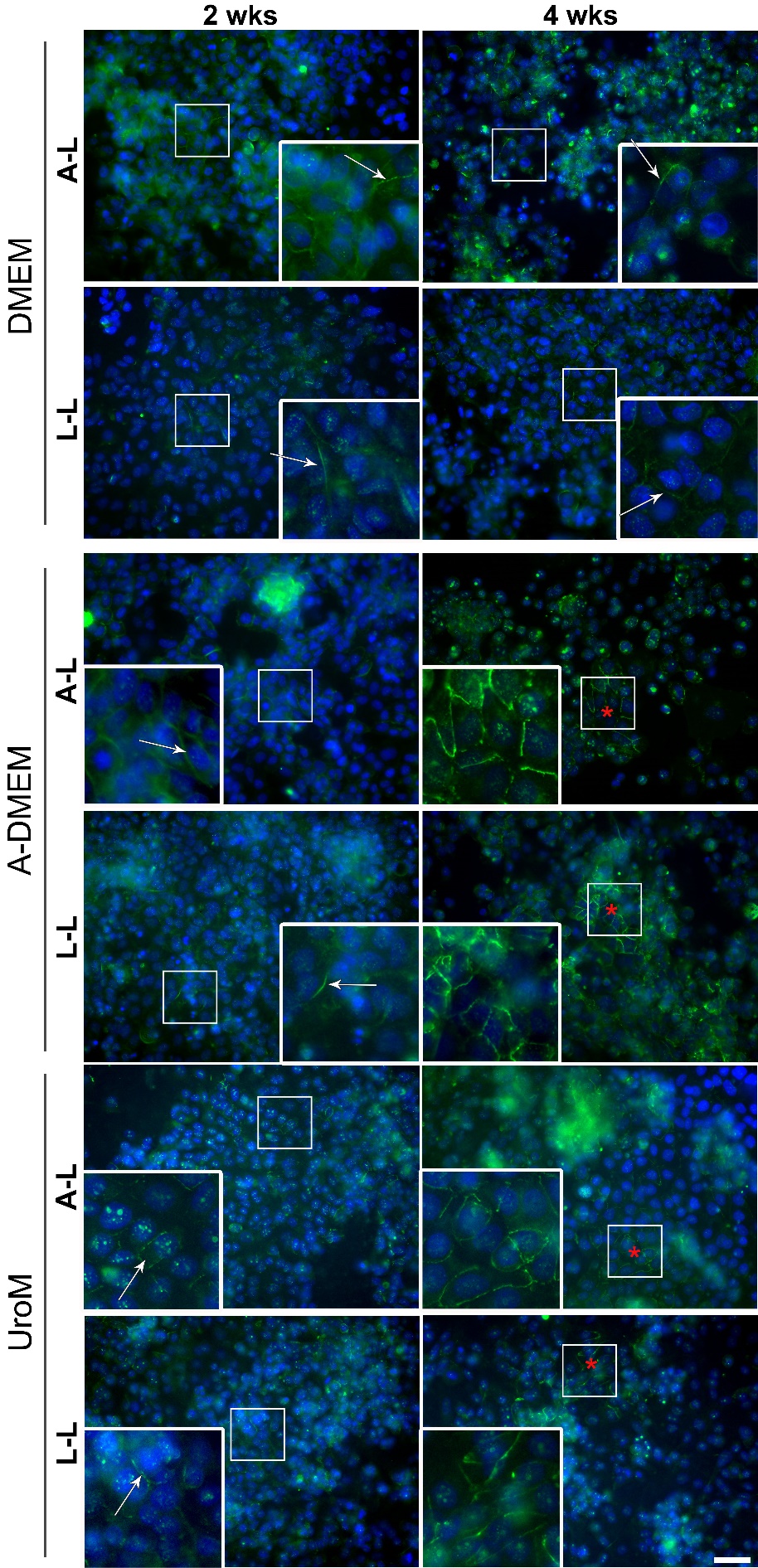


**Supplemental Figure S5**. Immunofluorescence of occludin (green) of FLO-1 cells grown on porous membranes in DMEM, A-DMEM and UroM culture media at the A-L and L-L interface for 2 or 4 wks. Nuclei are blue (DAPI). At 2 wks of culturing occludin is rarely present at lateral borders of neighbouring cells (arrows). At 4 wks of culturing occludin expression is high in some culture areas in A-DMEM and UroM media (areas indicated by red asterisks). Large insets framed with *white* lines are 150% enlarged images of corresponding small white framed insets. Scale bar, 50 µm.

**
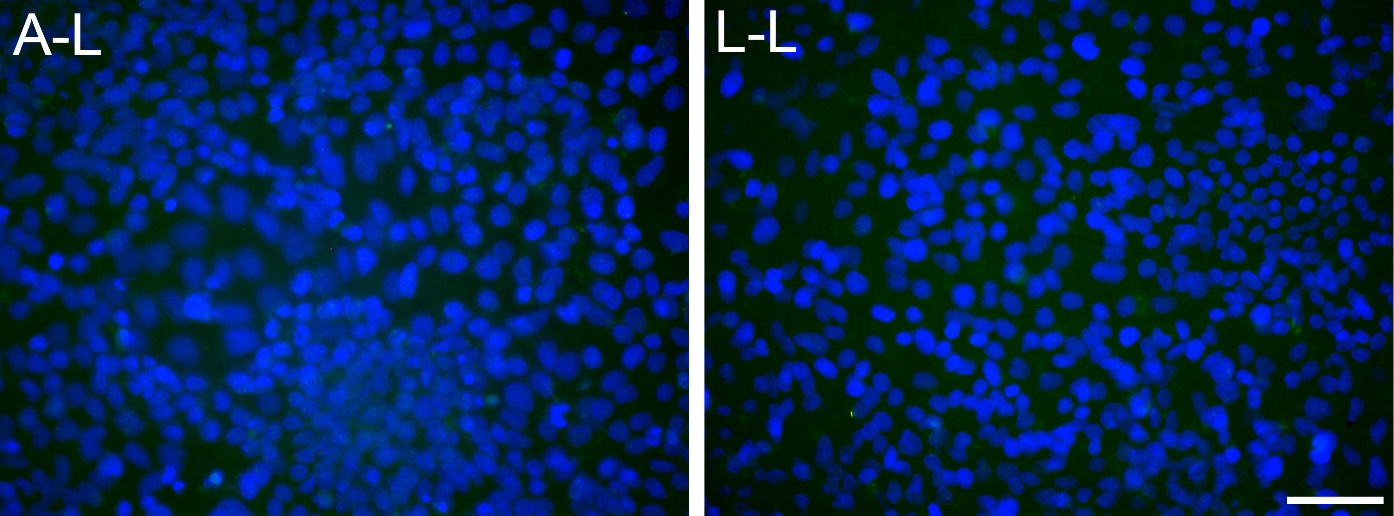
**

**Supplemental Figure S6.** Immunofluorescence of claudin-8 in FLO-1 cells grown on porous membranes in A-DMEM medium at the A-L and L-L interface for 1 wk. FLO-1 cells do not express claudin-8. Nuclei are stained blue. Scale bar, 50 µm.


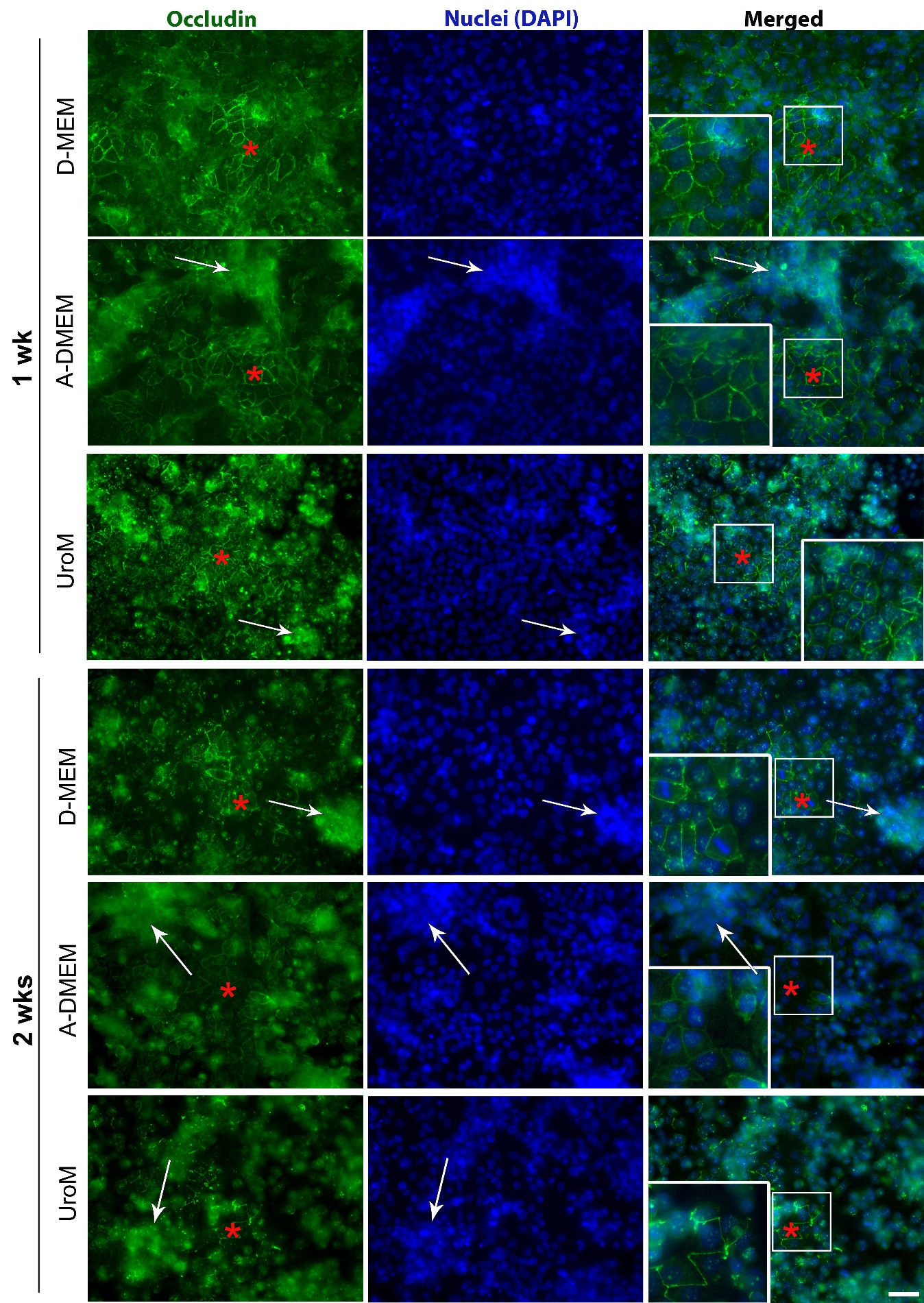


**Supplemental Figure S7**. Immunofluorescence of occludin (green) in submerged FLO-1 models. FLO-1 cells were maintained in DMEM, A-DMEM or UroM medium for 1 and 2 weeks. Red asterisks indicate areas with high occluding expression (at lateral plasma membrane). The expression of occludin is low in cells forming spheres, indicated by arrows. Large insets framed with *white* lines are 100% enlarged images of corresponding small white framed insets. Scale bar, 50 µm.

**
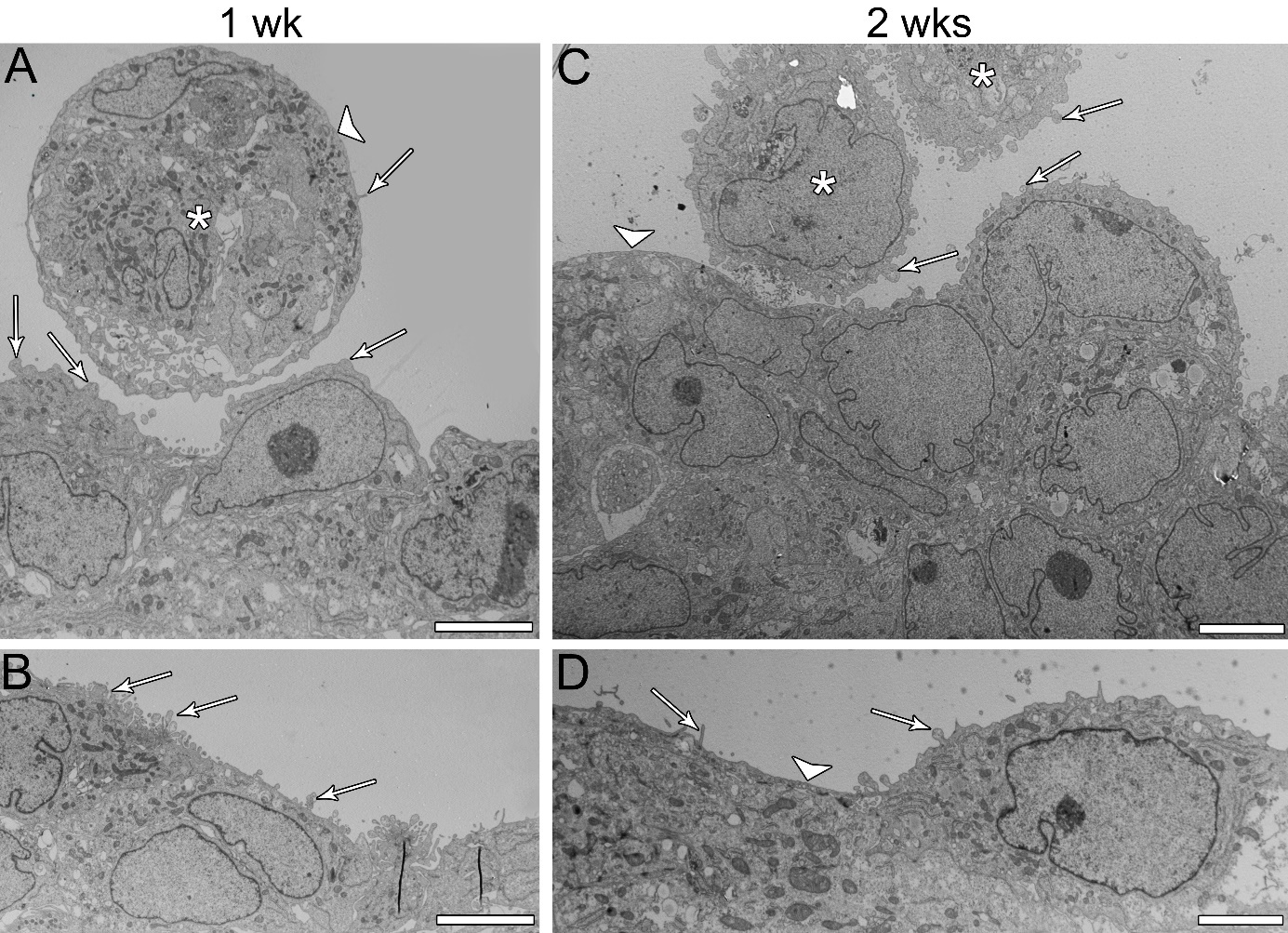
**

**Supplemental Figure S8.** The heterogeneity of cell shape and surface of submerged FLO-1 cell culture maintained in DMEM medium for 1 wk (A-B) and 2 wks (C-D). Cells form clusters (A, C) or monolayered/bilayered cultures (B, D). The cell surface is relatively smooth (arrowheads) or shaped in microvilli and/or round protrusions (arrows). Nuclei are large and irregularly shaped. Asterisks indicate cells loosely attached to neighbouring cells. Scale bars, 6 µm.

**
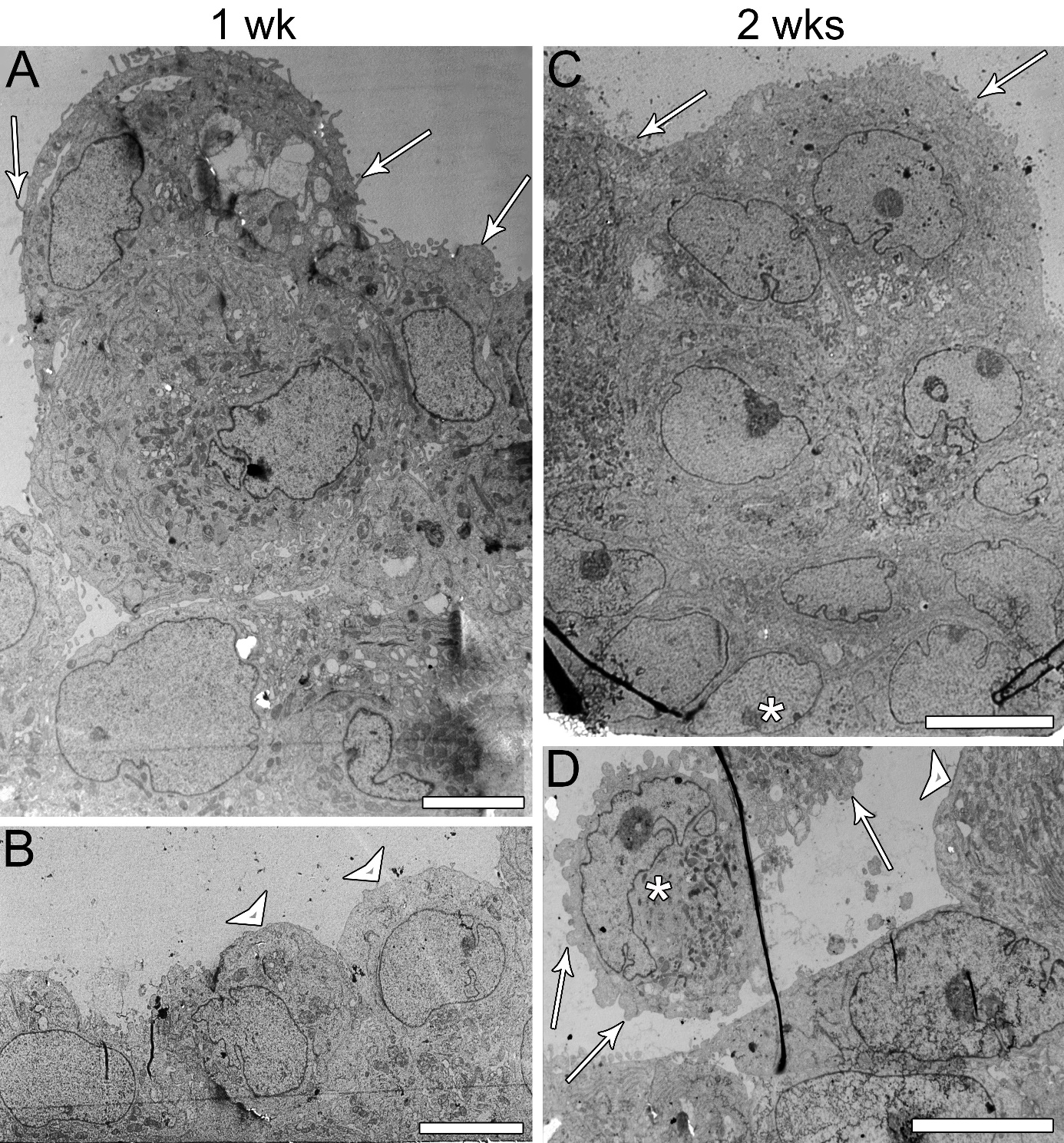
**

**Supplemental Figure S9.** The heterogeneity of cell shape and surface of submerged FLO-1 cell culture maintained in A-DMEM medium for 1 wk (A-B) and 2 wks (C-D). Cells form clusters (A, C) or mono/bilayer (B, D). The cell surface is relatively smooth (arrowheads) or shaped in microvilli and/or round protrusions (arrows). Nuclei are large. Asterisks indicate cells loosely attached to neighbouring cells. Scale bars, 6 µm (A), 8 µm (B, D), 10 µm (C).


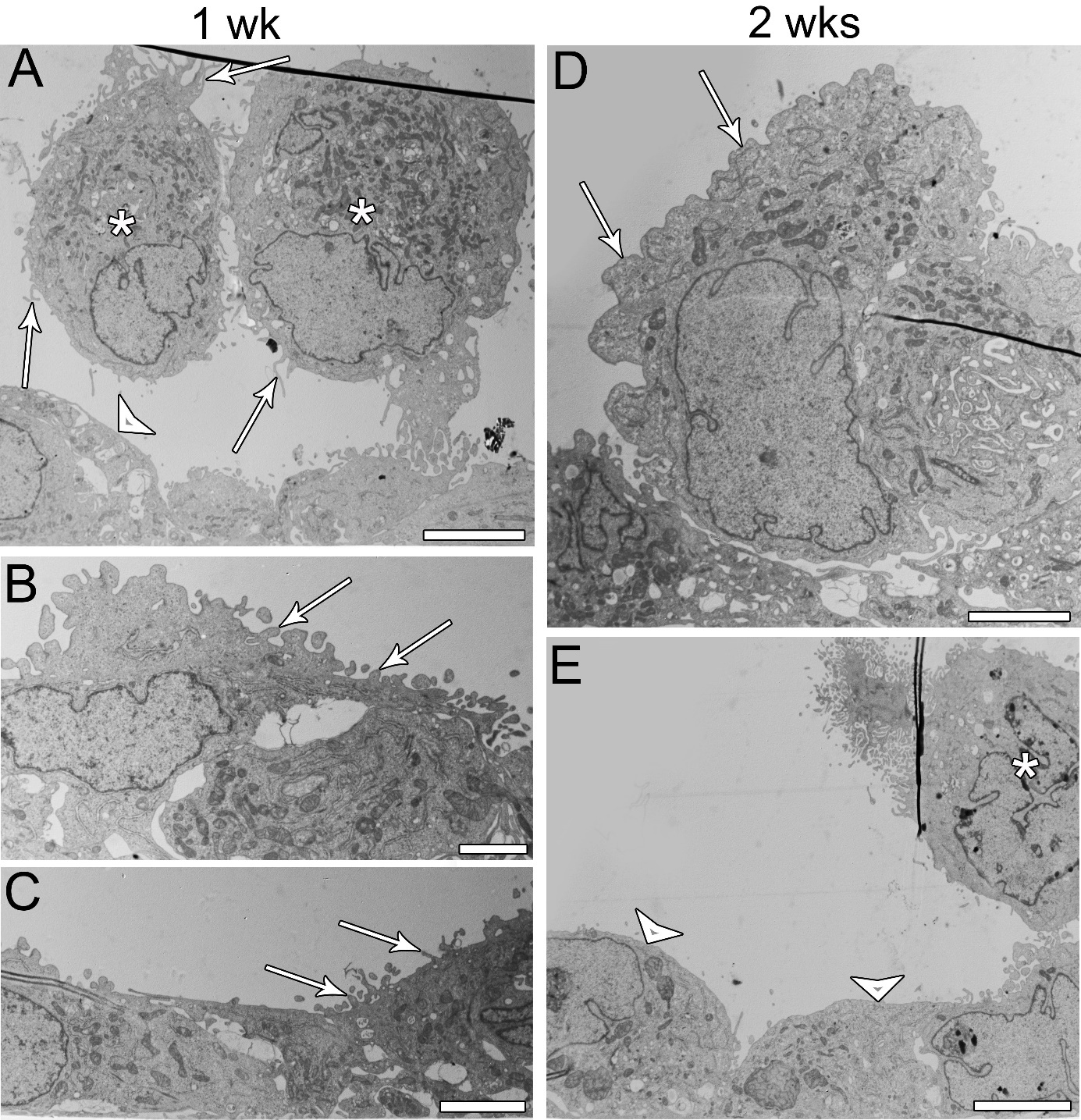


**Supplemental Figure S10.** The heterogeneity of cell shape and surface of submerged FLO-1 cell culture maintained in UroM medium for 1 wk (A-C) and 2 wks (D-E). Cells form clusters (A, D) or mono/bilayer (B, C, E). The cell surface is relatively smooth (arrowheads) or shaped in microvilli and/or round protrusions (arrows). Nuclei are large and irregularly shaped. Asterisks indicate cells loosely attached to neighbouring cells. Scale bars, 2 µm (B), 4 µm (C), 6 µm (A, D, E).

**Supplementary Table:**

Supplemental Table 1. Apparent permeability coefficients (P_app_s) of tested model drugs with high, moderate and low permeability in the short-term FLO-1 models cultured in DMEM or in A-DMEM.

| Model drug | FLO-1 (DMEM) | | FLO-1 (A-DMEM) | |
| --- | --- | --- | --- | --- |
|  | P_app A-B_ ± SE  (× 10^−5^ cm/s) | P_app B-A_ ± SE  (× 10^−5^ cm/s) | P_app A-B_ ± SE  (× 10^−5^ cm/s) | P_app B-A_ ± SE  (× 10^−5^ cm/s) |
| Propranolol | 2.35 ± 0.16^a^ | 2.04 ± 0.09^a^ | / | / |
| Antipyrine | 2.85 ± 0.05^b^ | 2.48 ± 0.10^b^ | 2.78 ± 0.06^a^ | 2.33 ± 0.02^a^ |
| Metoprolol | 1.32 ± 0.08^a^ | 1.76 ± 0.03^a^ | / | / |
| Losartan | 1.26 ± 0.07^b^ | 1.21 ± 0.01^b^ | 0.90 ± 0.06^a^ | 1.18 ± 0.05^a^ |
| Furosemide | 1.33 ± 0.03^a^ | 1.32 ± 0.02^a^ | / | / |
| Ranitidine | 1.67 ± 0.02^b^ | 1.36 ± 0.05^b^ | 1.26 ± 0.02^a^ | 1.13 ± 0.02^a^ |
| Atenolol | 1.26 ± 0.06^a^ | 1.41 ± 0.02^a^ | / | / |
| Famotidine | 1.51 ± 0.08^a^ | 1.49 ± 0.04^a^ | / | / |
| Oxacillin | 1.54 ± 0.05^b^ | 1.12 ± 0.05^b^ | 1.17 ± 0.08^a^ | 0.94 ± 0.01^a^ |
| Nadolol | 1.28 ± 0.02^a^ | 1.37 ± 0.09^a^ | / | / |
| Chlorothiazide | 1.67 ± 0.05^a^ | 1.42 ± 0.02^b^ | 1.40 ± 0.04^a^ | 1.61 ± 0.04^a^ |
| Azilsartan | 1.56 ± 0.07^a^ | 1.18 ± 0.06^a^ | 1.01 ± 0.02^a^ | 1.05 ± 0.01^a^ |

^a^n = 3

^b^n = 4
